# Developmental analysis of Spalt function in the *Drosophila* prothoracic gland

**DOI:** 10.1242/dev.202751

**Published:** 2024-08-27

**Authors:** Cristina M. Ostalé, Diego Pulido, Patricia Vega-Cuesta, Ana López-Varea, Jose F. de Celis

**Affiliations:** Centro de Biología Molecular ‘Severo Ochoa’, CSIC and Universidad Autónoma de Madrid, Madrid 28049, Spain

**Keywords:** Spalt, Prothoracic gland, Ecdysone, Nuclear lamina, Nucleolus

## Abstract

The Spalt transcriptional regulators participate in a variety of cell fate specification processes during development, regulating transcription through interactions with DNA AT-rich regions. Spalt proteins also bind to heterochromatic regions, and some of their effects require interactions with the NuRD chromatin remodeling and deacetylase complex. Most of the biological roles of Spalt proteins have been characterized in diploid cells engaged in cell proliferation. Here, we address the function of *Drosophila* Spalt genes in the development of a larval tissue formed by polyploid cells, the prothoracic gland, the cells of which undergo several rounds of DNA replication without mitosis during larval development. We show that prothoracic glands depleted of Spalt expression display severe changes in the size of the nucleolus, the morphology of the nuclear envelope and the disposition of the chromatin within the nucleus, leading to a failure in the synthesis of ecdysone. We propose that loss of ecdysone production in the prothoracic gland of Spalt mutants is primarily caused by defects in nuclear pore complex function that occur as a consequence of faulty interactions between heterochromatic regions and the nuclear envelope.

## Teaser

**Summary:** Loss of Spalt expression in the *Drosophila* prothoracic gland causes reduced ecdysone production, abnormalities in nucleolar size and nuclear membrane morphology, and reduced ERK phosphorylation and nuclear import.

## INTRODUCTION

The Spalt transcription factors Spalt major (Salm) and Spalt related (Salr) are conserved nuclear proteins bearing several pairs of C2H2 zinc fingers and a glutamine-rich region (de Celis and Barrio, 2009). These proteins participate in multitude of developmental processes acting as transcriptional repressors (de Celis and Barrio, 2009; Sánchez et al., 2011; Netzer et al., 2001). In the case of human Spalt-like proteins (SALL), this function is, in part, mediated through interactions with the histone deacetylase complex NuRD (Basta et al., 2017; Ma et al., 2018). *Drosophila* Salm and Salr can also act as transcriptional repressors in cultured cells and *in vivo*, although it appears that only Salr function depends on histone deacetylase activity (Sánchez et al., 2011). The SALL proteins also show multiple relationships with heterochromatin. Thus, *Drosophila* Salm binds preferentially heterochromatic regions (Ostalé et al., 2024) and human SALL1 and SALL4 proteins are preferentially located at pericentromeric heterochromatin (Netzer et al., 2006; Yamashita et al., 2007). Furthermore, SALL4 promotes the formation of heterochromatin in cancer cells (Kong et al., 2021). In this manner, there are several mechanisms of transcriptional repression mediated by Spalt proteins, which include interactions with histone modifiers such as NuRD (Lauberth and Rauchman, 2006) or the Lysine Demetylase 1 LSD1 (Liu et al., 2013), or association with heterochromatic regions, where SALL function could be involved in maintaining gene repression and general genome topology (Penagos-Puig and Furlan-Magaril, 2020).

The functions of Spalt proteins have been characterized mostly in diploid cells engaged in cell proliferation. However, the *Drosophila* genes are also expressed in some tissues formed by polyploid cells, such as the salivary glands and the prothoracic gland (PG). Polyploidy poses challenges to the regulation of gene expression, chromosomal stability, DNA replication and nuclear envelope function, and, because of their large nuclear size and resistance to apoptotic signals, polyploid cells constitute a convenient system for analyzing the inter-relationships between gene expression and nuclear chromatin organization. Furthermore, the cells of the PG produce ecdysone (Yamanaka et al., 2013), which plays a key role in the regulation of developmental transitions (Garen et al., 1977). The development and physiology of the PG and ecdysone biosynthesis are relatively well understood (Yamanaka et al., 2013; Ou et al., 2016), and many phenotypes associated with the loss ecdysone signaling have already been characterized (Mirth et al., 2005; Yamanaka et al., 2013; Kamiyama and Niwa, 2022). For these reasons, we have explored the roles of *Drosophila* Spalt proteins in the development and function of the PG.

The determination of ecdysone levels in the organism is subjected to multiple levels of regulation (Yamanaka et al., 2013). The main mechanisms regulating the levels of circulating ecdysone impinge on the expression of the enzymes that participate in the metabolic pathway that converts cholesterol into ecdysone in the PG (Kamiyama and Niwa, 2022). These proteins display monooxygenase activity (Phantom, Disembodied and Shadow; Feyereisen, 2012) and other enzymatic activities, such as those of glutathione transferase (Noppera-bo; Enya et al., 2014), the Rieske electron carrier (Neverland; Yoshiyama et al., 2006) and short-chain dehydrogenase/reductase (Shroud; Niwa et al., 2010), and are encoded by a group of genes named the Halloween genes (Rewitz et al., 2006; Gilbert et al., 2009). The expression of at least three of these genes in the PG, *phantom* (*phtm*; Cyp306A1), *disembodied* (*dib*; Cyp302A1) and *shadow* (*sad*; Cyp315A1), correlates with the titer of ecdysone in the hemolymph, suggesting that transcriptional activation of Halloween genes promotes the rises in ecdysone levels that trigger molting and pupariation (Rewitz et al., 2006).

The PG also integrates multiple signals from neurosecretory neurons and peripheral tissues through the use of signaling pathways such as Insulin, Target of Rapamacin (Layalle et al., 2008), Ras/ERK (Caldwell et al., 2005; Pan and O'Connor, 2021), Hippo (Moeller et al., 2017), Activin (Gibbens et al., 2011), BMP (Setiawan et al., 2018) and nitric oxide (Cáceres et al., 2011), which converge on the regulation of the expression of different Halloween genes (Yamanaka et al., 2013; Ou et al., 2016; Kamiyama and Niwa, 2022). In addition, a large group of transcription factors regulate the expression of different of Halloween gene. This set of transcription factors includes several C2H2 zinc-finger proteins, such as Ouija board (Komura-Kawa et al., 2015; Uryu et al., 2018), Molting defective (Neubueser et al., 2005; Danielsen et al., 2014) and Séance (Uryu et al., 2018). Interestingly, these three proteins cooperatively regulate the expression of only two Halloween genes, *neverland* and *spok*, which are localized in regions of pericentric heterochromatin (Fitzpatrick et al., 2011).

We show here that *salm* and *salr* (hereafter *salm/salr*) functions are required in the PG to promote the synthesis of ecdysone. In this regard, Spalt proteins behave in a similar manner to other transcriptional regulators of ecdysone biosynthesis, including Without children (Warren et al., 2001), Ventral veins lacking; (Danielsen et al., 2014), Ouija Board (Komura-Kawa et al., 2015) and Knirps (Danielsen et al., 2014). We also identify a prominent phenotype of Spalt mutant PG that affects the nuclear distribution of chromatin in these cells, the size of the nucleolus and the morphology of the nuclear envelope. Finally, we show that Spalt mutant PG have impaired ERK signaling, due to reduced ERK phosphorylation and nuclear import. We suggest that these phenotypes are connected, and correspond to a function of Salm/Salr in the maintenance of genome architecture within the nucleus. Failures in this organization lead to alterations in nuclear envelope and nuclear pore complex function, causing reduced ERK signaling and lower levels of Phantom (Phtm), Spookier (Spok), Disembodied (Dib) and Neverland (Nvd) expression. As a consequence of lower than required levels of ERK signaling, Spalt mutant PGs fail to produce the rise in the levels of ecdysone synthesis that trigger developmental transitions.

## RESULTS

### The function of *salm* and *salr* is required in the prothoracic gland for ecdysone synthesis

**Fig. 1.**
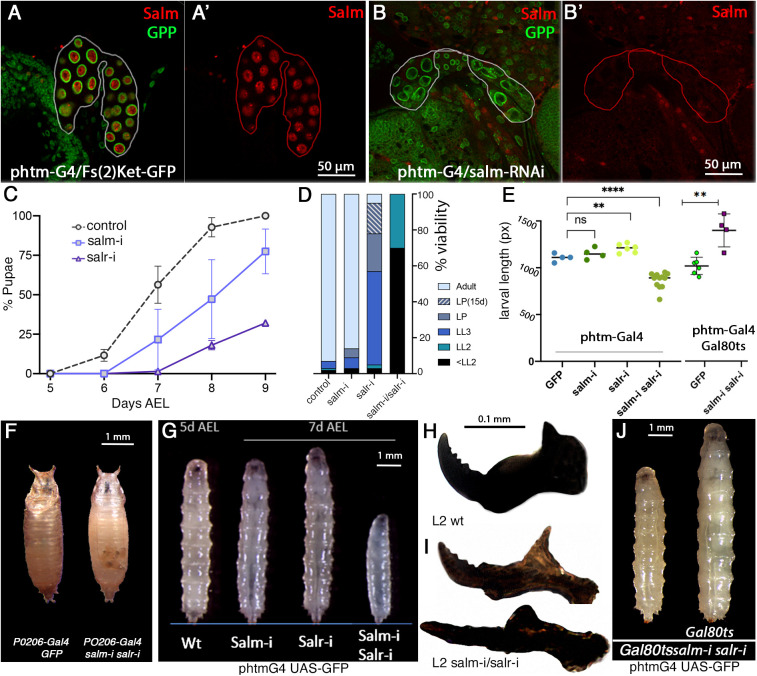
**Expression of Salm and phenotype of *salm*/*salr* knockdowns in the PG.** (A,A′) Expression of Salm (red) and Fs(2)Ket-GFP (green; nuclear membranes) in *UAS-Fs(2)ket-GFP/+; phtm-Gal4/+* PG. (B,B′) Expression of Salm (red) and Fs(2)ket-GFP in *UAS-Fs(2)ket-GFP/+; phtm-Gal4/UAS-salm-RNAi* PG. The periphery of the PG is delineated by a white or red line. (C) Percentage of larvae entering pupariation in three replicates at 5 to 9 days AEL in *phtm-Gal4 UAS-GFP/+* (control, circles), *UAS-salm-RNAi/+; phtm-Gal4 UAS-GFP/+*; (salm-i, squares) and *phtm-Gal4 UAS-GFP/UAS-salr-RNAi* (salr-i; triangles). Data are mean±s.d. (D) Percentage of survival at different developmental stages of *phtm-Gal4 UAS-GFP/+* (control), *UAS-salm-RNAi/+; phtm-Gal4 UAS-GFP/+*; (salm-i), *phtm-Gal4 UAS-GFP/UAS-salr-RNAi* (salr-i) and *UAS-salm-RNAi/+; phtm-Gal4 UAS-GFP/UAS-salr-RNAi* (salm-i/salr-i) knockdowns at the following stages: late L2 larvae (LL2, green), late LIII larvae (LL3, blue), pupal stage after 9 days AEL (LP, grey), pupal stage after 15 days AEL [LP(15d)] and adults. (E) Larval length (pixels) in controls (*phtm-Gal4 UAS-GFP/UAS-GFP*; GFP) and in *UAS-salm-RNAi/+; phtm-Gal4 UAS-GFP/+* (salm-i), *phtm-Gal4 UAS-GFP/UAS-salr-RNAi* (salr-i), *UAS-salm-RNAi/+; phtm-Gal4 UAS-GFP/UAS-salr-RNAi* (salm-i salr-i), *tub-Gal80^ts^/UAS-GFP; phtm-Gal4 UAS-GFP/+* (GFP) and *tub-Gal80^ts^/UAS-salm-RNAi; phtm-Gal4 UAS-GFP/UAS-salr-RNAi* (salm-i salr-i). Data are mean±s.d. **P<0.01, ****P<0.0001 (paired *t*-test). (F) Early wild-type pupa (*P0206-Gal4/UAS-GFP*; left panel) and early lethal *salm-i/salr-i* pupa (*P0206-Gal4/UAS-salm-RNAi; UAS-salr-RNAi/+*: right panel). (G) Differences in larval size in the following genotypes: *phtm-Gal4 UAS-GFP/+* (Wt) at 5 days after egg laying (5d AEL), and in *UAS-salm-RNAi/+; phtm-Gal4 UAS-GFP/+* (Salm-i), *phtm-Gal4 UAS-GFP/UAS-salr-RNAi* (Salr-i) and *UAS-salm-RNAi/+; phtm-Gal4 UAS-GFP/UAS-salr-RNAi* (Salm-i/Salr-i) 7 days after egg laying (7 days AEL). (H) Larval mouth hooks of wild-type L2 larvae (L2 wt). (I) Representative examples of *phtm-Gal4 UAS-GFP/UAS-salr-RNAi* larval mouth hooks (L2 salm-i/salr-i) that are curved (top) or straight (bottom). (J) Larval size in *UAS-salm-RNAi/tub-Gal80^ts^; phtm-Gal4 UAS-GFP/UAS-salr-RNAi* (Gal80ts/salm-i/salr-i; right) compared with controls *tub-Gal80^ts^/UAS-GFP; phtm-Gal4 UAS-GFP/+* (Gal80ts; left).

The Spalt transcription factors Salm and Salr are expressed in the PG (Fig. 1A,A′), and this expression depends on regulatory regions located between the *salm* and *salr* genes (Barrio et al., 1999). To identify a requirement for Spalt function in this tissue, we expressed RNAi directed against *salm* or *salr*, or against both genes in the PG in *phtm-Gal4 UAS-GFP/UAS-RNAi* flies. The expression of *salm-RNAi* (*salm-i*) reduces Salm nuclear expression in the PG (Fig. 1B,B′). *Salm* knocked-down larvae (*phtm-Gal4 UAS-GFP/UAS-salm-i*) progress with normal frequencies throughout the larval stages and pupal development (Fig. 1C,D), but show a weak delay in pupariation (Fig. 1C,D). When the expression of *salr* is knockdown (*salr-i*; *phtm-Gal4 UAS-GFP; UAS-salr-i/+*), the phenotype is stronger and only 24% of mutant larvae enter pupariation in the first 9 days AEL (Fig. 1D). This number increases to 43% after 15 days AEL (Fig. 1D), and the remaining *phtm-Gal4 UAS-GFP/UAS-salr-i* larvae display a developmental arrest in late L3 (Fig. 1D). Most *phtm-Gal4 UAS-GFP/UAS-salr-i* pupae die during pupal development (5% of adults; Fig. 1D).

Larvae in which both *salm* and *salr* expression are knocked down (*phtm-Gal4 UAS-GFP/UAS-salm-i; UAS-salr-i/+*) show larval lethality in L1 and L2 (Fig. 1D), and the 30% of surviving L2 larvae display a fully penetrant developmental arrest as late L2 larvae (Fig. 1D,G-I). Larval size is not affected by loss of *salm*, it is slightly increased in *salr* knockdowns, and strongly reduced in the double *salm/salr* knockdown (Fig. 1E,G). When we reduced the efficiency of the double *salm/salr* knockdown, by using the *P0206-Gal4* and *amnC651-Gal4* drivers or the *tub-Gal80^ts^* construct, we observed an array of phenotypes, including early pupal lethality (*P0206-Gal4/UAS-salm-i; UAS-salr-i/+*; Fig. 1F), a combination of smaller (54%) and larger than normal (46%) L3 larvae (*amnC651-Gal4; UAS-salm-i/+; UAS-salr-i*), and a fully penetrant formation of larger than normal L3 larvae that cannot progress to pupariation (*tub-Gal80^ts^/UAS-salm-i; phtm-Gal4/UAS-salr-i*; Fig. 1J). These larvae remain in an extended third instar stage for up to 20 days, and reach a larval size larger than controls (Fig. 1E,J). Both the strong and weak phenotypes of *salm*/*salr* knockdowns in the PG are compatible with defects in the synthesis or secretion of ecdysone by this endocrine organ (Yamanaka et al., 2013).

**Fig. 2.**
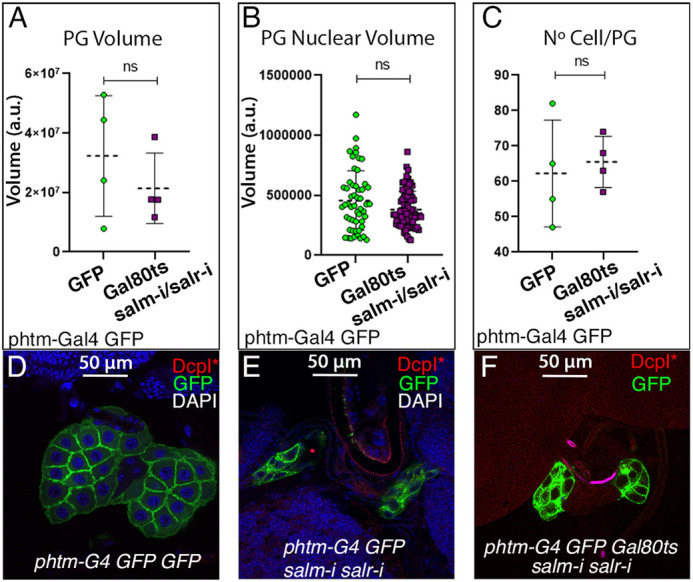
**Developmental parameters of *salm/salr* mutant prothoracic glands.** (A) PG volume (length × width × height in pixels) in controls (*tub-Gal80^ts^/UAS-GFP; phtm-Gal4 UAS-GFP/+*; GFP) and in *salm/salr* knockdowns (*tub-Gal80^ts^/UAS-salm-RNAi/+; phtm-Gal4 UAS-GFP/UAS-salr-RNAi*; Gal80ts/salm-i/salr-i). (B) PG nuclear volume (length × width × height in pixels) in controls (*phtm-Gal4 tub-Gal80ts/UAS-GFP*; GFP) and in *salm/salr* knockdowns (*tub-Gal80^ts^/UAS-salm-RNAi; phtm-Gal4 UAS-GFP/UAS-salr-RNAi*; Gal80ts/salm-i/salr-i). (C) Average number of cells in control PG (*tub-Gal80^ts^/UAS-GFP; phtm-Gal4 UAS-GFP/+*; GFP) and in *salm/salr* knockdowns (*tub-Gal80^ts^/UAS-salm-RNAi; phtm-Gal4 UAS-GFP/UAS-salr-RNAi*; Gal80ts/salm-i/salr-i). Data are mean±s.d. ns, not significant (paired *t*-test). (D-F) Expression of cleaved DcpI (DcpI*) in control PG (*phtm-Gal4 UAS-GFP/+*; D), and in the strong (*UAS-salm-RNAi/+; phtm-Gal4 UAS-GFP/UAS-salr-RNAi*, E) or weak (*tub-Gal80^ts^/UAS-salm-RNAi; phtm-Gal4 UAS-GFP/UAS-salr-RNA* larvae grown for 2 days at 25°C, for 5 days at 17°C and for 5 days at 29°C; F) knockdown conditions. The expression of GFP is in green; expression of DAPI is in blue in D and E.

We searched for changes in the size, number of cells and cell viability in the PG of *tub-Gal80^ts^/UAS-salm-i; phtm-Gal4/UAS-salr-i* individuals, but could not find any change in these general biological parameters in this genotype that prevents pupariation (Fig. 2A-C). The PG is reduced in size in *salm*/*salr* stronger knockdown conditions (*UAS-salm-i /+; phtm-Gal4 UAS-GFP/UAS-salr-i*; Fig. 2D,E), suggesting a role for these proteins in the regulation of the normal development of the PG. In genotypes where the morphology and size of the PG is not compromised but still cause a failure to progress to pupariation, the function of Salm/Salr is more likely to be related to the synthesis or secretion of ecdysone by morphologically normal PGs.

**Fig. 3.**
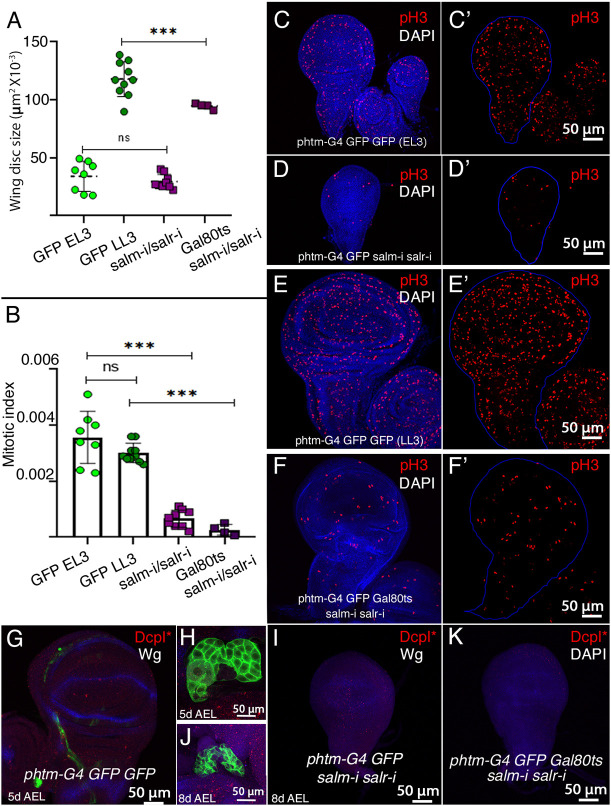
**Wing imaginal disc phenotype of *salm/salr* knockdowns in the prothoracic gland.** (A) Wing disc size in early L3 (GFP EL3; 90 h AEL) and late L3 (GFP LL3; 120 h AEL) *phtm-Gal4 UAS-GFP/+* wing discs compared with *UAS-salm-RNAi/+; phtm-Gal4 UAS-GFP/UAS-salr-RNAi* (salm-i/salr-i) and *tub-Gal80^ts^ UAS-salm-RNAi; phtm-Gal4 UAS-GFP/UAS-salr-RNAi* (Gal80ts/salm-i/salr-i) wing discs from larvae grown for 2 days at 25°C, for 5 days at 17°C and for 5 days at 29°C. All values are in pixels ×10^−3^. (B) Mitotic index of early L3 (GFP EL3; 90 h AEL) and late L3 (GFP LL3; 120 h AEL) control wing discs of *phtm-Gal4 UAS-GFP/+* genotype, compared with the mitotic index of *UAS-salm-RNAi/+; phtm-Gal4 UAS-GFP/UAS-salr-RNAi* (salm-i/salr-i) and *tub-Gal80^ts^/+; UAS-salm-RNAi/+; phtm-Gal4 UAS-GFP/UAS-salr-RNAi* (Gal80ts/salm-i/salr-i). Data are mean±s.d. ***P<0.001 (paired *t*-test). (C-E′) Examples of early (C) and late L3 (E) wing discs showing the expression of phospho-Histone 3 (pH3; red) and DAPI (blue) in control discs (*phtm-Gal4 UAS-GFP/UAS-GFP*; C,E) and *salm/salr* knockdowns (*UAS-salm-RNAi/+; phtm-Gal4 UAS-GFP/UAS-salr-RNAi*; D). (F) *tub-Gal80^ts^/+; UAS-salm-RNAi/+; phtm-Gal4 UAS-GFP/UAS-salr-RNAi* wing discs from larvae grown for 2 days at 25°C, for 5 days at 17°C and for 5 days at 29°C. Independent red channels are shown in C′-F′, and the outline of the wing discs is shown in blue in C′-F′. (G-K) Expression of active DcpI (red) reveals apoptotic cells in control discs (*phtm-Gal4 UAS-GFP/UAS-GFP*; G) and in *salm/salr* knockdown discs of *UAS-salm-RNAi/+; phtm-Gal4 UAS-GFP/UAS-salr-RNAi* (H) and *tub-Gal80^ts^/+; UAS-salm-RNAi/+; phtm-Gal4 UAS-GFP/UAS-salr-RNAi* (K) genotypes. The expression of Wingless (Wg; blue) is shown in G and I, and the corresponding PG of control discs and *salm/salr* knockdown discs are shown in H and J.

To confirm a deficiency in ecdysone signaling, we analyzed different readouts associated with loss of ecdysone in larval tissues. First, we monitored cell division in the wing disc, as progression through the cell cycle in the imaginal epithelium depends on ecdysone during the third larval instar (Herboso et al., 2015). We found that the imaginal discs of arrested late L2 *UAS-salm-i /+; phtm-Gal4 UAS-GFP/UAS-salr-i* larvae (25°C) reach a maximal size similar to that of early L3 wild-type discs (Fig. 3A,C-D). In *tub-Gal80^ts^/UAS-salm-i; phtm-Gal4/UAS-salr-i* late third instar larvae (2 days at 25°C, 5 days at 17°C and 5 days at 29°C) the wing discs are smaller than normal (Fig. 3A,E-F). To identify mitotic cells, we used an antibody that recognizes histone 3 phosphorylated in Ser10 (pH3, Fig. 3C-F′). For comparison between discs of different sizes, we calculated the mitotic index (the ratio between the number of mitotic cells and the disc area). The mitotic index has an approximately constant value in wild-type discs during L3 development (Fig. 3B). Both *salm/salr* silenced conditions led to a strong reduction in the mitotic index (Fig. 3B,C-F′). As we failed to observe an increment in the presence of apoptotic cells in these wing discs (Fig. 3G-K), the reduction in disc size is mostly caused by a failure of imaginal cells to enter mitosis.

**Fig. 4.**
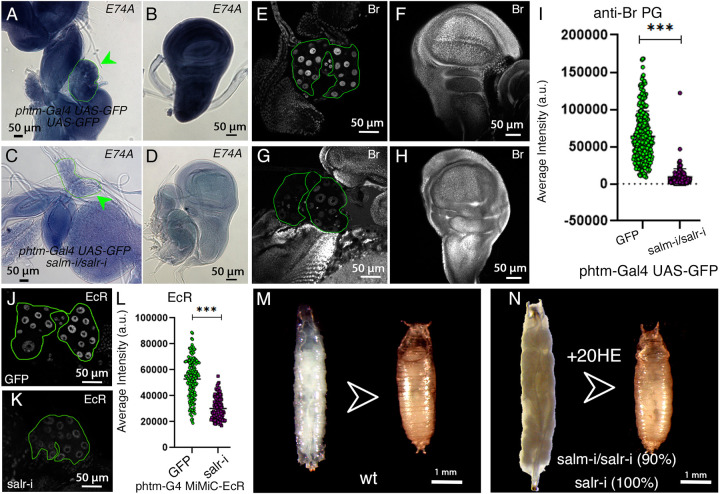
**Expression of ecdysone signaling target genes in *salm/salr* knockdowns in the prothoracic gland.** (A,B) mRNA expression of *E74A* in wild-type PG (A; green arrowhead) and in the wing imaginal disc (B). (C,D) mRNA expression of *E74A* in *UAS-salm-RNAi/+; phtm-Gal4 UAS-GFP/UAS-salr-RNAi* PG (C; green arrowhead) and in the wing imaginal disc (D). (E,F) Expression of Broad protein (Br) in wild-type PG (E) and in the wing imaginal disc (F). (G,H) Expression of Br in *UAS-salm-RNAi/+; phtm-Gal4 UAS-GFP/UAS-salr-RNAi* PG (G) and in the wing imaginal disc (H). The PG is delineated with a green line in A, C, E and G. Wing discs and PG were dissected from 6-7 day AEL larvae. (I) Average intensity of Broad protein expression in the PG of control (*phtm-Gal4 UAS-GFP/+*; green circles) and *UAS-salm-RNAi/+; phtm-Gal4 UAS-GFP/UAS-salr-RNAi* (salm-i/salr-I; purple circles). Data are mean±s.d. ***P<0.001 (paired *t*-test). (J,K) Expression of MiMIC-EcR in the PG of control (*phtm-Gal4 UAS-GFP*; J) and *phtm-Gal4 UAS-GFP/UAS-salr-RNAi* PG (salr-i; K). (L) Average intensity of MiMIC-EcR protein expression in the PG of control (*phtm-Gal4 UAS-GFP*; green circles) and *phtm-Gal4 UAS-GFP/UAS-salr-RNAi* (salr-i; purple circles). Data are mean±s.d. ***P<0.001 (paired *t*-test). (M) Normal transition from L3 larvae to early pupa in wild type. (N) Transition from L3 larvae to early pupa in tubGal80^ts^/+; *UAS-salm-RNAi/+; phtm-Gal4 UAS-GFP/UAS-salr-RNAi* larvae treated with 1 mg/ml 20hydroxyecdysone (20HE) at 5 days AEL. The frequency of pupariation rescue is indicated as a percentage for each genotype.

As a second readout of ecdysone signaling, we monitored the expression of transcriptional targets of the hormone in different tissues. The expression of Ecdysone-induced protein 74A (E74A) mRNA is reduced in all larval tissues after *salm-*RNAi and/or *salr-*RNAi knockdown in the PG (Fig. 4A-D). The expression of Broad protein (Br) is also reduced in this genotype, but the reduction is observed only in cells of the PG (Fig. 4E-I). Finally, we also studied the expression of the gene encoding the ecdysone receptor (EcR) using the MiMIC strain EcR-3xFLAG-GFP. The expression of the EcR in the PG is regulated by ecdysone signaling, constituting a positive feedback loop that leads to an increase in the synthesis of ecdysone by PG cells before the larval-to-pupal transition (Shirai et al., 2012). We found a substantial reduction of EcR expression in the PG after knockdown of *salr* (Fig. 4J-L). Altogether, these results suggest that ecdysone signaling is defective in the PG and in peripheral tissues in larvae with a reduction in *salm/salr* expression in the PG.

The phenotype of *salm/salr* knockdown in the PG is reminiscent to that of several other transcription factors that have been analyzed using either mutant alleles or knockdown conditions. Thus, *loss of vvl, kni, woc* or *ouib* function in the PG prevent the larval to pupal transition, and these phenotypes are rescued by dietary exposure to 20-hydroxyecdysone (20HE; Neubueser et al., 2005; Danielsen et al., 2014; Niwa and Niwa, 2016; Uryu et al., 2018; Zhao et al., 2021). We found that 20HE supplemented in the fly food rescues cell proliferation in the wing disc (0.05 mg/ml 20HE; Fig. S1), and promotes the pupariation of *UAS-salm-i/tub-Gal80^ts^; phtm-Gal4/UAS-salr-i* (90%, 0.5 mg/ml 20HE; Fig. 4M,N) and *phtm-Gal4 UAS-GFP/UAS-salr-i* larvae (100%, 0.5 mg/ml 20HE). *salm/salr* and *salr* knockdown larvae forced to enter into pupariation by exogenous ecdysone die soon after they form the pupal case (Fig. 4N).

### The expression of Halloween genes is reduced in *salr* knockdown prothoracic glands

**Fig. 5.**
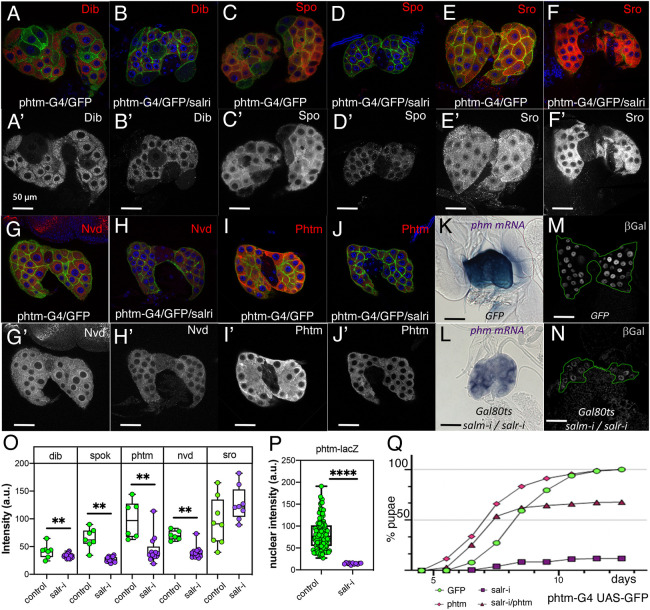
**Expression of the Halloween genes *dib*, *spo*, *sro*, *nvd* and *phtm* in *salr*, and in *salm*/*salr* knockdowns in the prothoracic gland.** (A,B) Expression of Dib in control (A; *phtm-Gal4 UAS-GFP/+*) and *phtm-Gal4 UAS-GFP/UAS-salr-RNAi* (B; phtm-G4/GFP/salr-i) PGs. (C,D) Expression of Spo (Spo) in control (C; *phtm-Gal4 UAS-GFP/+*) and *phtm-Gal4 UAS-GFP/UAS-salr-RNAi* (D; phtm-G4/GFP/salr-i). (E,F) Expression of Sro (Sro) in control (E; *phtm-Gal4 UAS-GFP/+*) and *phtm-Gal4 UAS-GFP/UAS-salr-RNAi* (F; phtm-G4/GFP/salr-i). (G,H) Expression of Nvd in control (G; *phtm-Gal4 UAS-GFP/+*) and *phtm-Gal4 UAS-GFP/UAS-salr-RNAi* (H; phtm-G4/GFP/salr-i). (I,J) Expression of Phtm protein (Phtm) in control (I; *phtm-Gal4 UAS-GFP/+*) and *phtm-Gal4 UAS-GFP/UAS-salr-RNAi* (J; phtm-G4/GFP/salr-i). The expression of each protein is shown in red, and the corresponding red channels are shown in A′-J′. The expression of GFP is in green and the expression of DAPI in blue. All PG were dissected from larvae at 7 days AEL. (K,L) Expression of *phtm* mRNA in control *tub-Gal80^ts^/+; phtm-Gal4 UAS-GFP/UAS-GFP* (GFP; K) and in *tub-Gal80^ts^/+; UAS-salm-RNAi/+; phtm-Gal4 UAS-GFP/UAS-salr-RNAi* (L; *Gal80^ts^/salm-i/salr-i*) PGs. (M,N) Expression of *phtm-lacZ* in control (M; *phtm-LacZ/tubGal80^ts^; phtm-Gal4 UAS-GFP/UAS-GFP*) and in *phtm-LacZ/tubGal80^ts^; UAS-salm-RNAi/+; phtm-Gal4/UAS-salr-RNAi* (N). All larvae of combinations involving *tubGal80^ts^* were grown for 2 days at 25°C, for 5 days at 17°C and for 5 days at 29°C before dissection. (O) Quantification of fluorescence intensity levels of Dib, Spok, Phtm, Nvd and Sro protein expression in control (*phtm-Gal4 UAS-GFP/+*; green circles) and in *phtm-Gal4 UAS-GFP/UAS-salr-RNAi* (salr-i; purple circles) PGs. Data are median, interquartile range and maximal and minimal values. **P<0.01 (paired *t*-test). (P) Quantification of fluorescence intensity levels of βGal in control (*tubGal80^ts^*/*phtm-lacZ; phtm-Gal4 UAS-GFP/+;* GFP, green circles) and in *tubGal80^ts^*/*phtm-lacZ; UAS-salm-RNAi/+; phtm-Gal4 UAS-GFP/UAS-salr-RNAi* (Gal80ts salm-i/salr-i; purple squares) PGs. Data are median, interquartile range and maximal and minimal values. ****P<0.0001 (*t*-test). (Q) Percentage of pupae at different days AEL from larvae of the following genotypes: *phtm-Gal4 UAS-GFP/UAS-GFP* (GFP; green circles), *UAS-pthm*/+; *pthm-Gal4 UAS-GFP/+* (pink diamonds), *phtm-Gal4 UAS-GFP/UAS-salr-RNAi* (purple squares) and *UAS-pthm*/+; *phtm-Gal4/UAS-salr-RNAi* (purple diamonds).

Transcriptional regulation of the Halloween genes is central to regulating the production of ecdysone in the PG. We studied in control and *salr* knockdown PGs the expression of *disembodied* (*dib*), *spook* (*spo*), *neverland* (*nvd*), *shroud* (*sro*) and *phantom* (*phtm*) (Fig. 5). The expression of Dib (Fig. 5A-B,O), Spo (Fig. 5C-D,O), Nvd (Fig. 5G-H,O) and Phtm (Fig. 5I-J,O) is reduced in 7-day-old larvae of *phtm-Gal4 UAS-GFP/UAS-salr-RNAi* PG compared with their controls *phtm-Gal4 UAS-GFP/+*, and only the expression of Sro is not modified in *salr* knockdown PG compared with their controls (Fig. 5E-F,O). We also observed a reduction in the expression of *phtm* mRNA (Fig. 5K,L) in the PG of *tub-Gal80^ts^; UAS-salm-i/+; phtm-Gal4 UAS-GFP/UAS-salr-i* larvae, and this reduction was confirmed using the reporter construct *phtm-lacZ* by monitoring β-galactosidase expression (Fig. 5M-N,P). We tried rescue experiments by expressing *nvd* (*UAS-nvd-HA/+; phtm-Gal4/UAS-salr-*i) or *phtm* (*UAS-phtm/+; phtm-Gal4/UAS-salr-*i) in *salr-*RNAi knockdown larvae. In the case of *nvd* we did not find a rescue of pupariation in salr-RNAi larvae (32% of pupae compared with 26% in *phtm-Gal4 UAS-GFP/UAS-salr-*i). In contrast, ectopic expression of *phtm* (*UAS-phtm/+; phtm-Gal4 UAS-GFP/UAS-salr-*i) is able to rescue to a large extent pupariation in *salr* knockdown larvae (Fig. 5Q), indicating that some of the effects of Salr insufficiency could be caused by reduced *phtm* expression.

### Chromosomal organization, nuclear morphology and nucleolar size are strongly affected in *salm/salr* knockdown prothoracic glands

**Fig. 6.**
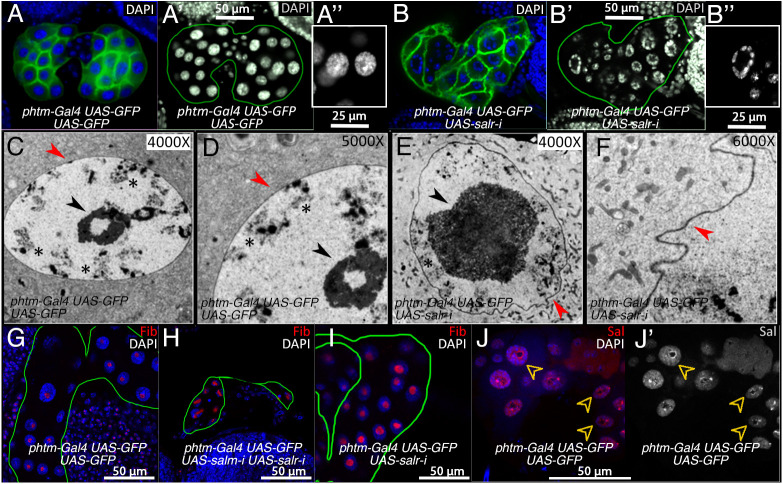
**Alterations in nuclear morphology, nucleolar size and chromatin disposition in *salr* and *salm/salr* knockdowns in the prothoracic gland.** (A-A″) Expression of CD8-GFP in the PG of *phtm-Gal4 UAS-GFP/UAS-GFP* PG stained with DAPI to reveal nuclear DNA. (B-B″) Expression of CD8-GFP in the PG of *phtm-Gal4/UAS-GFP/UAS-salr-RNAi* PG stained with DAPI. A′ and B′ correspond to DAPI expression; A″ and B″ correspond to higher magnifications (2×) of normal and *salr* knockdown nuclei, respectively. (C,D) TEM images of PG nuclei showing the normal morphology of the nucleolus and nuclear envelope. (E,F) TEM images of *salr* knockdown nuclei showing the enlarged nucleolar size (E) and the convoluted appearance of the nuclear envelope (F). The nucleolus is indicated by a black arrowhead, the nuclear membrane by red arrowheads and the chromosomes by asterisks. (G) Expression of Fibrillarin (Fib, red channel) in control *phtm-Gal4 UAS-GFP/UAS-GFP* PG. (H) Expression of Fibrillarin (Fib, red channel) in *UAS-salm-RNAi/+; phtm-Gal4 UAS-GFP/UAS-salr-RNAi* PG. (I) Expression of Fibrillarin (Fib, red channel) in *phtm-Gal4 UAS-GFP/UAS-salr-RNAi* PG. (J,J′) Expression of Salm (Sal, red channel) in *phtm-Gal4 UAS-GFP/UAS-GFP* PG. DAPI is shown in blue in G-J. Arrowheads indicate nuclei with Salm expression around the nucleolus.

So far, the phenotypes displayed by loss of Salm/Salr in the PG are compatible with a role for these proteins in the regulation of the expression of a subset of Halloween genes, and this function might imply a function of Salm/Salr as direct transcriptional regulators. Such a role has been postulated for other transcription factors (Kamiyama and Niwa, 2022). However, we noticed that *salm/salr* mutant PGs also display changes in subcellular morphology that might be related to a more general failure in the physiology of these cells. To analyze in more detail these alterations, we focused on the knockdown of *salr*, because in this genetic background (*phtm-Gal4 UAS-GFP/UAS-salr-i*) the insufficiency in ecdysone production affects the transition from the larval stage to pupariation; however, the overall morphology of the PG of mutant larvae is similar to that of wild-type larvae (Fig. 6A-B).

The main subcellular changes that we could identify in *salr* knockdown PGs were alterations in the disposition of the DNA within the nucleus, as well as changes in the morphology of the nuclear envelope and the size of the nucleolus. Thus, the DNA of *salr-*RNAi mutant PGs appears displaced towards the periphery of the nucleus (Fig. 6B-B″), occupying most of the nuclear volume in normal PGs (Fig. 6A-A″). The change in DNA nuclear disposition is accompanied by an increase in the size of the nucleolus (Fig. 6C-E, black arrowheads). This increase in size is apparent in TEM pictures (compare Fig. 6C with E), and is also revealed by the expression of Fibrillarin (Fib; Fig. 6G-I), a ribosomal pre-RNA processing protein localized in the nucleolus (Rodriguez-Corona et al., 2015). These nuclear phenotypes are also observed after knockdown of *salm* and *salr* in the PG (Fig. 6H). Interestingly, the accumulation of Salm within the nucleus of the PG in mature L3 larvae is maximal in the periphery of the nucleolus (Fig. 6J,J′). Finally, we also observed by TEM a loss of circularity in the nuclear envelope in *salr* knockdowns (Fig. 6C-F, red arrowheads). Thus, the nuclear envelope is now undulated and with multiple indentations (Fig. 6F, red arrowheads). All these phenotypes indicate a relationship between Sal proteins and both the nuclear envelope and the nucleolus.

**Fig. 7.**
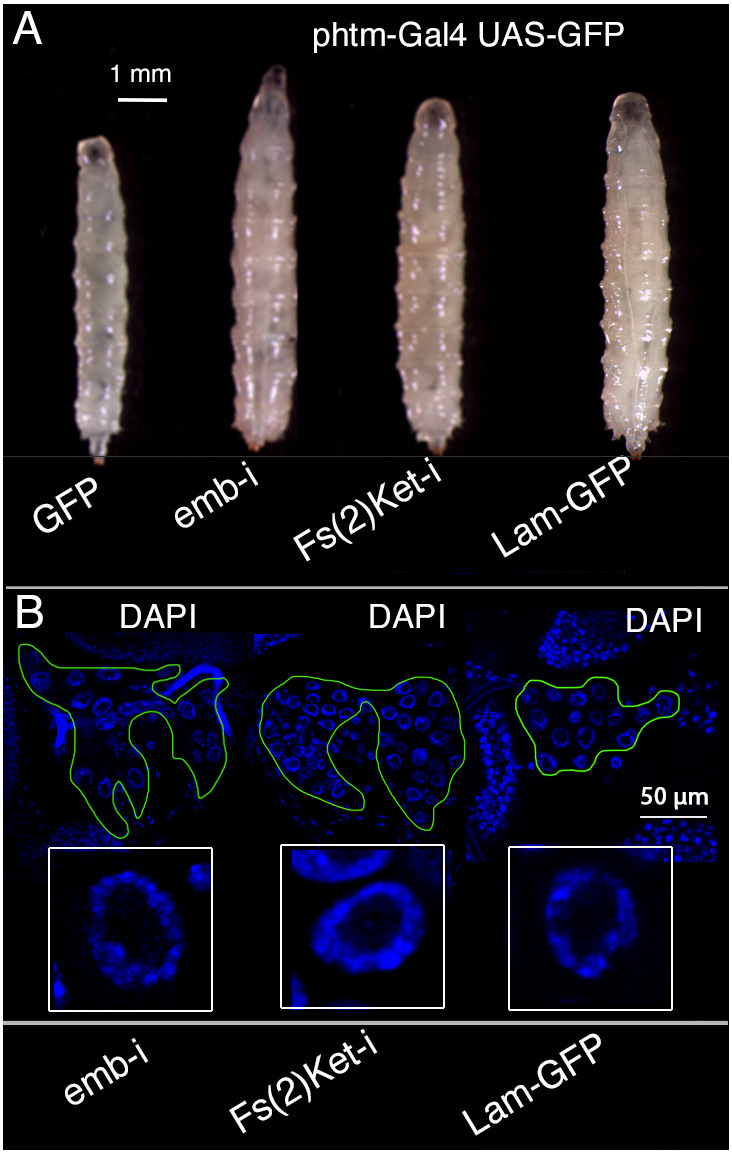
**Larval phenotypes and chromatin disposition in the prothoracic gland of genetic alterations in genes affecting the nuclear lamina.** (A) Five-day-old control larva (GFP; *phtm-Gal4 UAS-GFP/UAS-GFP*, left) and 7-day-old larvae of *phtm-Gal4 UAS-GFP/UAS-RNAi* [*emb-i* and *Fs(2)Ket-i*] and *phtm-Gal4 UAS-GFP/UAS-Lam-GFP* (Lam-GFP) genotypes. Expression of *embargoed* RNAi (emb-i) and *Female sterle 2 of Ketel* [Fs(2)Ke-i] in the PG prevents pupariation in a similar manner to overexpression of Lamin-GFP (*phtm-Gal4/UAS-Lamin-GFP*; Lam-GFP). (B) Morphological appearance of the PG (delineated with a green line) of larvae with the same genotypes as in A, and higher magnification of the nuclei stained with DAPI (blue) showing the circular and peripheral appearance of the chromatin.

Changes in nucleolar size, nuclear membrane morphology and nuclear distribution of chromatin have been described in different tissues and in cultured cells upon alterations in the expression of the genes Lamin and LaminC (Buchwalter and Hetzer, 2017; Karoutas and Akhtar, 2021), encoding the *Drosophila* members of vertebrate Lamin B and Lamin A (Pałka et al., 2018). Lamins are the main constituents of the nuclear lamina, a filamentous network located at the nuclear and nucleolar periphery that determines the mechanical characteristics of the nucleus, and is related to the nucleus-cytoplasmic transport machinery and the three-dimensional organization of the chromatin within the nucleus (Adam et al., 2008; Karoutas and Akhtar, 2021; Petrovsky et al., 2018; Xie et al., 2016). To search for similarities between the effects of *salm/salr* loss of function conditions in the PG on nucleolar size and chromosomal organization, we characterized in the PG the consequences of loss of function conditions of genes directly related to the organization of the nuclear envelope. We find that knockdown of the exportin *embargoed* (*emb*) or the importin *Female sterile (2) Ketel* [*Fs(2)Ket*] in the PG prevent pupariation and result in L3 larvae of larger size and extended development (Fig. 7A). These proteins are involved in the assembly of the nuclear lamina and the export and import of proteins through the nuclear pore complex (Adam et al., 2008; Xie et al., 2016). Similarly, overexpression of Lamin (Lamin-GFP), which has a disorganizing effect on the nuclear lamina (Petrovsky et al., 2018), also results in a similar phenotype of extended L3 development and larger larval size (Fig. 7A). Furthermore, the chromatin of these individuals shows an abnormal distribution towards the nucleus periphery (Fig. 7B), forming a ring-shaped disposition similar to that observed upon loss of *salm/salr* in the PG.

### Loss of Sal function disrupts MAPK/ERK signaling

The MAPK/ERK signaling pathway plays a key role in the regulation of ecdysone synthesis, and it is activated by different tyrosine kinase receptors (Cruz et al., 2020; Pan and O′Connor, 2021; Rewitz et al., 2009). The state of activation of the MAPK/ERK pathway can be visualized by the accumulation of the di-phosphorylated form of ERK (dpERK; Cruz et al., 2020), a protein that, upon activation, is imported into the nucleus through the nuclear pores in a process mediated by Importin7 and Fs(2)Ket (Lorenzen et al., 2001). Considering the effects of *salm/salr* knockdown on nuclear morphology, and the similarity of the *salm/salr* phenotype with loss of *emb* or *Fs(2)Ket*, and with Lamin overexpression, it is possible that the import of activated ERK into the nucleus is compromised in *salm/salr* loss-of-function conditions.

**Fig. 8.**
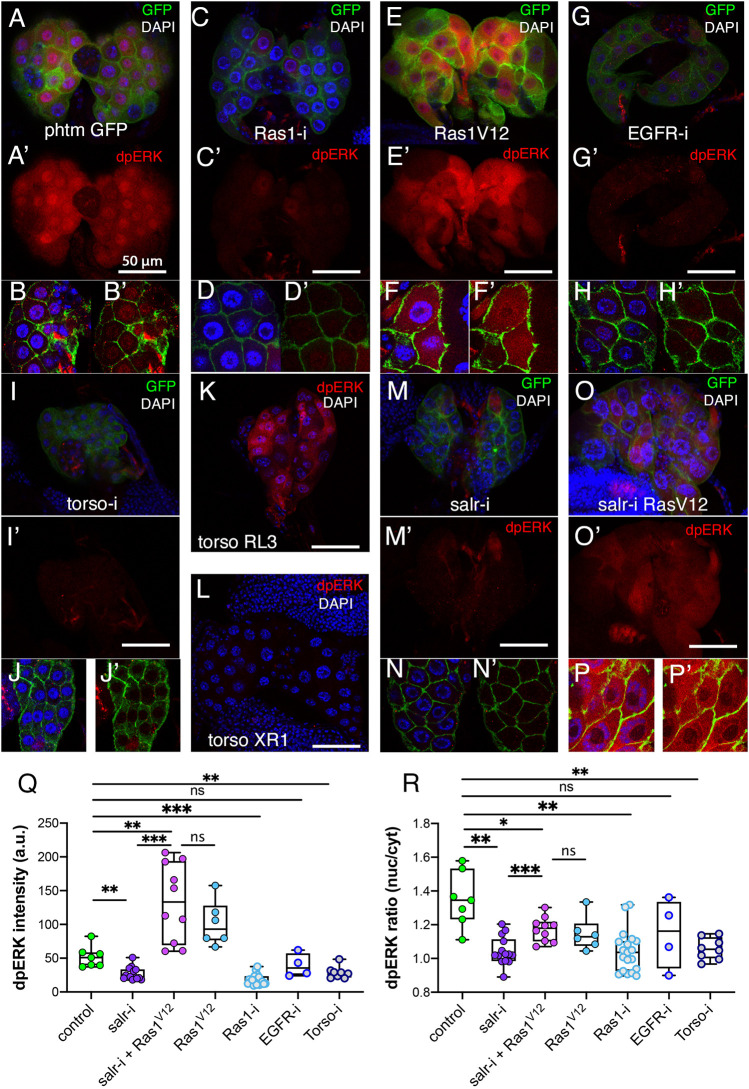
**Effects of *salr* knockdown on ERK activation and nuclear localization.** (A,B) Expression of dpERK (red) in the PG of 7-day-old control larvae (*phtm-Gal4 UAS-GFP/+*). (C-I) Expression of dpERK (red) in the PG of 7-day-old larvae expressing *UAS-Ras1-RNAi* (Ras1-i; C,D), *UAS-Ras1^V12^* (Ras1V12; E,F), *UAS-EGFR-RNAi* (EGFR-i; G,H) and *UAS-torso-RNAi* (torso-i, I,J) in *phtm-Gal4 UAS-GFP/UAS-RNAi* combinations. The images in B, D, F, H and I are at twice the magnification of those in A, C, E, G and I. The red channels of A, C, E, G and I are shown in A′, C′, E′, G′ and I′, and a composite of green and red channels is shown in B′, D′, F′, H′ and J′. (K,L) Expression of dpERK (red) in *torso* gain-of-function (*tor^RL3^*; K) and loss-of-function (*tor^XR1^*; L) alleles. The expression of DAPI is in blue. (M,N) Expression of dpERK (red) in *phtm-Gal4 UAS-GFP/UAS-salr-RNAi*. The individual red channel of M is shown in M′; N and N′ are pictures taken at twice magnification of M and M′. The red and green channels of N ae shown in N′. (O,P) Expression of dpERK (red) in *UAS-Ras1^V12^/+; phtm-Gal4 UAS-GFP/UAS-salr-RNAi*. The individual red channel of O is shown in O′; P and P′ are pictures taken at twice the magnification of O and O′. The red and green channels of P are shown in P′. (Q,R) Quantification of dpERK intensity levels (Q) and dpERK nucleus/cytoplasm intensity levels (R) measured in individual PGs extracted from larvae at 7 days AEL of the following genotypes: *phtm-Gal4 UAS-GFP/+* (control), *phtm-Gal4 UAS-GFP/UAS-salr-RNAi* (salr-i), *UAS-Ras1^V12^/+; phtm-Gal4 UAS-GFP/UAS-salr-RNAi* (salr-i+Ras1V12), *UAS-Ras1^V12^/+; phtm-Gal4 UAS-GFP/+* (Ras1^V12^), *phtm-Gal4 UAS-GFP/UAS-Ras1-RNAi* (Ras1-i), *phtm-Gal4 UAS-GFP/UAS-EGFR-RNAi* (EGFR-i) and *phtm-Gal4 UAS-GFP/UAS-torso-RNAi* (Torso-i). Data are median, interquartile range and maximal and minimal values. *P<0.05, **P<0.01, ***P<0.001 (paired *t*-test). ns, not significant.

The expression of dpERK is detected in the PG of late third instar larvae (Ohhara et al., 2015; Cruz et al., 2020), where dpERK accumulates in both the nucleus and cytoplasm (Fig. 8A-B,R). Consistent with previous observations (Cruz et al., 2020), we find that dpERK expression is very much reduced after knockdowns of *Ras1* (Fig. 8C-D,Q), *EGFR* (Fig. 8G-H,S) and *Torso* (Fig. 8I-J,S). Similarly, gain- and loss-of-function alleles of *torso* have opposite effects on dpERK accumulation (Fig. 8K-L). The levels of dpERK increase when an activated version of Ras1 (Ras1^V12^) is expressed in the PG (Fig. 8E-F,Q). These changes of dpERK expression are accompanied by a slight reduction in the nucleus-cytoplasm ratio of dP-ERK accumulation (Fig. 8R), and in no case is the expression of Salm affected in PG glands with alterations in Ras/ERK signaling (Figs S2 and S3).

**Fig. 9.**
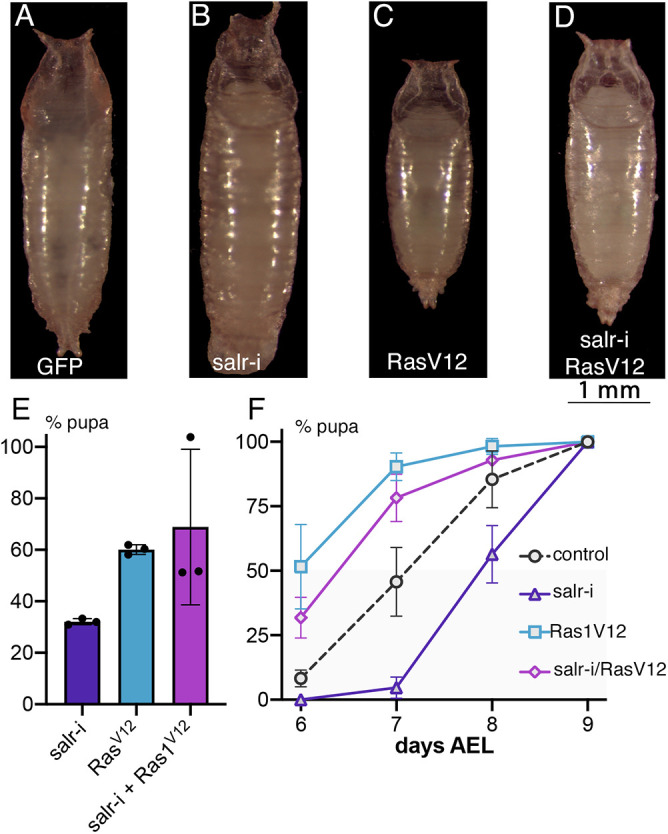
**Rescue of *salr* knockdowns by Ras1^V12^ overexpression.** (A-D) Representative pupae of *phtm-Gal4 UAS-GFP/+* (GFP; A), *phtm-Gal4 UAS-GFP/UAS-salr-RNAi* (salr-I; B), *UAS-Ras1^V12^/+; phtm-Gal4 UAS-GFP/+* (Ras1V12; C) and *UAS-Ras1^V12^/+; phtm-Gal4 UAS-GFP/UAS-salr-RNAi* (salr-i/Ras1V12; D). (E) Percentage of pupal formation at 9 days AEL in three replicates of larvae of *phtm-Gal4 UAS-GFP/UAS-salr-RNAi* (salr-i), *UAS-Ras1^V12^/+; phtm-Gal4 UAS-GFP/+* (Ras1^V12^) and *UAS-Ras1^V12^/+; phtm-Gal4 UAS-GFP/UAS-salr-RNAi* (salr-i+Ras1V12). Data are mean±s.d. (F) Daily frequency of pupariation at 6, 7, 8 and 9 days AEL expressed as a percentage of the total pupae formed at 9 days AEL in three replicates of larvae of *phtm-Gal4 UAS-GFP/+* (control), *phtm-Gal4 UAS-GFP/UAS-salr-RNAi* (salr-i), *UAS-Ras1^V12^/+; phtm-Gal4 UAS-GFP/+* (Ras1V12) and *UAS-Ras1^V12^/+; phtm-Gal4 UAS-GFP/UAS-salr-RNAi* (salr-i/RasV12). Data are mean±s.d.

Surprisingly, the knockdown of *salr* in the PG leads to a strong reduction in dpERK expression levels (Fig. 8M-L,Q), and in this case the nucleus-cytoplasm ratio of its accumulation is very much reduced (Fig. 8R). To better define the impact of *sal* function on ERK activation, we carried out a rescue experiment in a *salr* knockdown background using the expression of Ras1^V12^. We found that Ras1^V12^ can rescue to a large extent the loss of dpERK caused by loss of *salr* (Fig. 8O-P,Q-R), without affecting the increase in nucleolar size characteristic of *salr* mutant PG (Fig. 8M,O). The rescue of dpERK levels is accompanied by a phenotypic rescue of pupariation time and frequency of pupae (Fig. 9). In fact, the frequency of mutant pupae, as well as the pupariation time of *salr-*RNAi*/Ras1^V12^* larvae is very similar to that of *Ras1^V12^* larvae (Fig. 9E,F). Taken together, these results suggest that reduced ERK activation is a fundamental aspect of the *Spalt* loss-of-function phenotype in the PG.

We tried to carry out the converse experiments, i.e. rescue of Ras/ERK signaling by Salm/Salr overexpression. However, overexpression of Salm or Salr in the PG results in phenotypes that are reminiscent of loss of *salm/salr* function (Fig. S4), which we think are caused by a dominant-negative effect of these proteins when overexpressed. These phenotypes include the formation of small larvae (84%) and large L3 larvae with extended L3 development (16%). Only 17% of these large L3 larvae can enter pupariation, dying as early pupae. The corresponding PG of the LL3 larvae appears very much disorganized and contains cells of different sizes (Fig. S4).

To better visualize ERK nuclear accumulation in wild-type and *sal* mutant conditions, we expressed a tagged ERK protein (HA-ERK) in the PG. The overexpression of HA-ERK in the PG does not affect larval development or the larval-to-pupal transition, resulting in normal adult flies. In this background, we found that loss of *salr* consistently reduced dpERK expression (Fig. S5). The overexpression of HA-ERK did not modify the characteristic phenotype of *salr* knockdowns, and these larvae (*UAS-HA-ERK/+; phtm-Gal4 UAS-GFP/UAS-salr-RNAi*) remain arrested in L3 at the same frequencies as *phtm-Gal4 UAS-GFP/UAS-salr-RNAi* larvae. We also detected differences in the subcellular localization of HA-ERK in control and *salr* knockdown PGs. The localization of HA-ERK in control PGs shows a nuclear versus cytoplasmic ratio of 2:1 (Fig. S5). In contrast, this ratio is lower in *salr* knockdowns, reaching an average value of 1:1 (Fig. S5), similar to that observed in the case of *Fs(2)Ket* knockdown (Fig. S5). The function of the Fs(2)Ket β-Importin is required for ERK nuclear import (Lorenzen et al., 2001), and the similarity of the *Fs(2)Ket* and *salr* loss-of-function phenotypes suggests that nuclear import of ERK is compromised in the absence of Salr. The nuclear protein Osa, in contrast, is not affected by the reduction of Salr in the PG, and its localization is exclusively nuclear (Fig. S5). Altogether, these results suggest that Salr is required to reach maximal levels of ERK activation and to promote its internalization into the nucleus.

## DISCUSSION

We have characterized the consequences of *salm* and *salr* knockdowns in the larval PG. We find a variety of phenotypes affecting the size, morphology, subcellular characteristics and physiology of the PG, the larval tissue formed by polyploid cells where ecdysone synthesis and secretion takes place. The *salm/salr* phenotypic syndrome ultimately leads to the failure of mutant *salm/salr* larvae to enter pupariation, suggesting that the PG fails to produce enough levels of ecdysone at this crucial developmental transition. Thus, all the characteristic phenotypes of reduced ecdysone titer, including delayed pupariation, failures to progress from L2 to L3 or from L3 to pupal development, loss of mitotic imaginal cells and pupal lethality, are observed to different degrees upon *salm*, *salr* or both *salm* and *salr* knockdowns. Furthermore, the expression of several ecdysone signaling target genes is reduced in *salm/salr* knockdowns, and (as a minimum) the failure to develop from L3 larvae to pupae can be rescued by ecdysone supplemented in the diet. In this manner, our first conclusion is that Salm and Salr play a key role in regulating the synthesis of ecdysone by the PG. In genotypes with strong and simultaneous reduction of *salm* and *salr*, this function could be related to changes in the size or number of cells observed in mutant PG. In contrast, weaker genetic combinations display phenotypes of reduced ecdysone signaling in larvae with PGs of normal size.

### Complementarity of the Salm and Salr requirements

To what extent the genes *salm* and *salr* contribute to the same overall function in the PG? The Salm and Salr proteins have significant differences in sequence (Barrio et al., 1996) and also show distinct requirements for histone deacetylase complex activity for transcriptional repression (Sánchez et al., 2011). We find stronger effects in the case of *salr* versus *salm* knockdowns in the PG. These differences may be caused by different effectiveness of the RNAi lines used against each gene. Several arguments support some degree of independent requirements for each gene. Thus, individual knockdowns do show mutant phenotypes, which is a result not expected if Salm and Salr displayed complete redundancy. Furthermore, a reduction of only *salr* expression is able to reproduce most of the phenotypes observed in a weak double *salm* and *salr* knockdown, indicating a requirement for Salr that cannot be compensated for by the presence of Salm. Similarly, a reduction in only Salm causes a delay in pupariation time that can be interpreted as a consequence of a mild reduction of ecdysone production by the PG. These results should be contrasted with the stronger phenotype caused by the double *salm* and *salr* knockdown, consisting of the failure to progress to L3 that is never observed upon individual gene knockdowns.

Overall, our results suggest that Salm and Salr regulate with a degree of independence or complementarity the same set of processes leading to normal ecdysone production. A model we think can accommodate these results implies the formation of homo- and heterodimers of Salm and Salr proteins. The existence of physical interactions of different Spalt proteins with each other has already been observed in several vertebrate models (Sweetman et al., 2003; Sakaki-Yumoto et al., 2006). If this was the case for the *Drosophila* proteins in the PG, we need only to assume different degrees of effectiveness of Salm and Salr homo- and heterodimers for the same molecular functions, with Salm homodimers showing lower activity than those including Salr. As the phenotypes we observe differ quantitatively more than qualitatively, for the rest of this discussion we shall refer to Salm/Salr function without distinguishing possible independent contributions of each individual protein.

### Possible mechanisms of Salm and Salr function in the prothoracic gland

Our work indicates that the Salm/Salr proteins belong to the group of transcription factors whose loss in the PG causes a delay in larval development, developmental arrests at the L1/L2, L2/L3 or L3/pupal transitions, or pupal lethality (Ou et al., 2016). These proteins constitute ∼15% of all transcription factors encoded in the *Drosophila* genome (Ou et al., 2016), indicating that ecdysone synthesis is under very rigorous control by proteins with the potential to act as transcriptional regulators. In some cases where the effects of these transcription factors were analyzed in more depth, it was found that the expression of some or all genes encoding the enzymes implicated in the ecdysone biosynthetic pathway was reduced (Danielsen et al., 2014). Furthermore, the Halloween genes *spok*, *phtm* and *dib* contain predicted binding sites for both Vvl and Kni, and these proteins can bind regulatory sites in the *phtm* gene (Danielsen et al., 2014). Paradoxically, some of these transcriptional regulators, including Kni and Sal, are transcriptional repressors (Arnosti et al., 1996; Sánchez et al., 2011), which implies that they are not direct regulators of Halloween genes. In this manner, a direct function of Salm/Salr on *phtm* transcriptional activation seems unlikely, even though *phtm* and other Halloween genes require Salm/Salr activity for their expression. Thus, we need to propose an alternative mechanism of Salm/Salr action by which these proteins could compromise the expression of Halloween genes in response to other signals playing a direct activating function. So far, our results are compatible with two scenarios that need not be mutually exclusive and that reconcile two of the main observations of our work: (1) loss of *salm/salr* affects chromosomal organization within the nucleus, and nucleolar size and nuclear membrane morphology; and (2) loss of *salm/salr* compromises ERK activation and its nuclear localization.

#### A role for Salm/Salr in chromatin organization

Sal proteins could play a role in the generation and maintenance of heterochromatin, and we have identified highly enriched binding of Salm to heterochromatic regions in ChIP-seq experiments (Ostalé et al., 2024). These observations indicate that *Drosophila* Sal proteins, which can act as canonical transcriptional repressors (Ostalé et al., 2024), could participate in the organization of the heterochromatin, a functional aspect that is conserved for vertebrate SALL proteins (Netzer et al., 2001, 2006; Yamashita et al., 2007). As the *salm/salr* mutant phenotype shares several characteristics with those caused by defects in the organization of the nuclear lamina (Bustin and Misteli, 2016; Penagos-Puig and Furlan-Magaril, 2020), we propose that one aspect of the *salm/salr* phenotype in the PG is caused by chromatin decompaction or by faulty interactions between heterochromatic regions and the lamina that surrounds the nuclear membrane and the nucleolus. It has been shown that alterations in heterochromatin-lamina interactions could lead to changes in the mechanical stiffness of the nuclear envelope, and could also alter the activity of the nuclear pore complex, leading to a misfunction of the nuclear import machinery (Bustin and Misteli, 2016).

#### A role for Salm/Salr in ERK activation

A second prominent effect of Salm/Salr loss that needs to be further explored relates to the defects we observe in ERK phosphorylation and nuclear import. ERK activation is a necessary step to burst the transcription of different Halloween genes and, consequently, is a crucial checkpoint for ecdysone production and the regulation of developmental transitions (Cruz et al., 2020; Pan and O'Connor, 2021; Rewitz et al., 2009). In this manner, the levels and timing of ERK activation by different tyrosine receptor kinases, and the feedback loops established in PG cells between ERK and ecdysone signaling, constitute a central node of the regulation of ecdysone production (Cruz et al., 2020; Pan and O'Connor, 2021; Queenan et al., 1997). We have observed defects both in ERK activation and ERK nuclear import in *salm/salr* and *salr* knockdowns that might be related to defects in nuclear pore complex assembly or function, processes to which the lamina and different importins contribute (Karoutas and Akhtar, 2021; Xie et al., 2016). Accordingly, we suggest that Salm/Salr function is required to preserve the integrity of the nuclear lamina and the operation of nuclear pore complexes, and that, as a consequence of defective activated ERK import into the nucleus, the levels of dp-ERK cannot reach the required threshold to activate the expression of Halloween genes or to engage in other positive-feedback loops with ecdysone signaling.

In summary, we propose that the effects of *salm/salr* loss in the PG are not related to a direct regulation of target genes involved in ecdysone synthesis, but to indirect effects of a general role of the Salm and Salr proteins in chromatin organization and heterochromatin interactions with the nuclear and nucleolar lamina. This function explains the profound alterations we observed in nuclear morphology, chromatin disposition and nucleolar size in *salm/salr* knockdown conditions. Interestingly, SALL1 accumulation is associated with the chromocenters at the nuclear envelope and at the periphery of the nucleolus in NIH-3T3-transfected cells (Netzer et al., 2001), suggesting a conserved role for Sal proteins in heterochromatin maintenance. We also propose that alterations in the lamina or lamina-heterochromatin interactions could lead to defects in nuclear import of ERK, preventing *salm/salr* knockdown cells to reach the levels of nuclear and phosphorylated ERK required to activate the expression of the Halloween genes. Although the scenario we propose is consistent with our observations, we are aware that we still lack a precise molecular mechanism to link Spalt function with chromatin structure, lamina organization and nuclear pore complex function. Such a mechanism might imply non-genetic functions of Spalt, e.g. a role as a bridge between heterochromatic DNA and the nuclear and nucleolar lamina, that deserve further analyses.

## MATERIALS AND METHODS

### *Drosophila* stocks and genetics

We used the *Gal4* lines *phtm-Gal4* (PG) and PO206-Gal4 (Mirth et al., 2005), *amnC651-Gal4* (Waddell et al., 2000), UAS-HA-ERK (Molnar and de Celis, 2013), *tub-Gal80^ts^*, *phtm-LacZ* (BL12198) and *EcR-3xFlag-GFP* (MiMIC, BL59823), and the following UAS strains: *UAS-nvd-Dm-HA* (Yoshiyama-Yanagawa et al., 2011), *UAS-lamin-GFP* (BL7376), *UAS-phtm* (Niwa et al., 2004), *UAS-salm-RNAi* (VDRC3029, BL60462), *UAS-salr-RNAi* (VDRC28386, BL29549), *UAS-Smt3-i* (Talamillo et al., 2008), *UAS-EcR-i* (BL9327), *UAS-emb-RNAi* (VDRC3347), *UAS-Fs(2)ket-RNAi* (VDRC22348), *UAS-Ras1^V12^* (Molnar and de Celis, 2013), *UAS-Ras1-RNAi* (NIG9375R), *UAS-EGFR-RNAi* (NIG10079R2), UAS-ERK-RNAi (GD4697) and *UAS-torso-RNAi* (GD2613). We also used the *torso* mutant alleles *tor^RL3^* (Sprenger et al., 1993) and *tor^XR1^* (Sprenger et al., 1989). Knockdown of Sal genes in combination with the *phtm-Gal4* driver were generated in the following genotypes: *UAS-salm-RNAi/+; phtm-Gal4 UAS-GFP/UAS-salr-RNAi* (salm-i/salr-i), *UAS-salm-RNAi/+; phtm-Gal4 UAS-GFP/+* (salm-i), *phtm-Gal4 UAS-GFP/UAS-salr-RNAi* (salr-i) and *tubGal80^ts^/+; UAS-salm-RNAi/+; phtm-Gal4 UAS-GFP/UAS-salr-RNAi* (Gal80ts salm-i/salr-i). This last genotype was grown for 2 days at 25° C, for 5 days at 17°C and for 5 days at 29°C before larval dissection.

### Prothoracic gland measurements and fluorescence intensity calculations

Nuclear volume (width×length×high) and number of cells in the PG were calculated from composite *z*-sections of PG mounted without squashing. Fluorescence intensity was calculated from images taken using a confocal microscope (LSM750; Zeiss) with the same laser intensity, pin hole, gain and speed settings. All calculations were made with Fiji (Image). All colocalization measures were made with the Coloc2 tool of Fiji to calculate the Pearson and Mander coefficients.

### Mouth hook dissection

The mouth hooks were dissected in water, removing the tissues attached to them with tweezers. After dissection, the mouth hooks were mounted in 70% glycerol in microscopic slices. The cover slides were sealed with clear nail polish, and the preparations photographed in a Zeiss Axioplan2 microscope using a Spot 2 digital camera.

### Mitotic index

We measured the wing blade area from composite *z*-sections (projections) of wing blade images taken using a confocal microscope (LSM750; Zeiss) and counted the number of phospho-Histone3 (pH3)-positive cells. The mitotic index was calculated as the ratio between pH3-positive cells and the wing blade area measured in pixels. Wing discs were taken from early L3 (EL3; 90±12 h AEL) and late L3 (LL3; 120±12 h AEL) larvae *of phtm-Gal4 UAS-GFP/UAS-GFP*, from late L2 *UAS-salm-RNAi/+; phtm-Gal4 UAS-GFP/UAS-salr-RNAi* (90±12 h AEL) and from late L3 *tubGal80^ts^/+; UAS-salm-RNAi/+; phtm-Gal4 UAS-GFP/UAS-salr-RNAi* larvae grown for 2 days at 25°C, for 5 days at 17°C and for 5 days at 29°C.

### *In situ* hybridization

RNA probes for *E74A* and *phtm* were made from 1 µg of cDNA clones RE03155 (pFLC1 vector; *phtm*) and SD03570 (pOT2 vector; *E74A*). The clones were digested for 2 h at 37°C with NotI (*phtm* antisense) and ApaI (*phtm* sense probe), and with XmnI (*E74A* antisense) and NotI (*E73A* sense). 200 ng of linearized DNA were incubated at 25°C for 4 h with the polymerases SP6 or T7 (*E74A* antisense and sense probes, respectively), or with T3 and T7 (*phtm* antisense and sense probes, respectively). Template DNA was digested with DNAaseI for 15 min at 37°C, and the probes were precipitated with LiCl 1 M and EDTA 0.5 M in absolute ethanol, and resuspended in water treated with diethyl pyrocarbonate (Sigma). *In situ* hybridization with sense and antisense probes was carried out as previously described (Organista et al., 2015). The prothoracic glands and imaginal discs were mounted in 70% glycerol and photographed with a Spot camera coupled to a Zeiss Axioplan microscope.

### Immunohistochemistry

We used rabbit and rat anti-Salm antibodies (Barrio et al., 1999), rabbit anti-phospho-Histone3, anti-diphosphorylated-Erk and anti-cleaved Cas3 (Cell Signaling Technology) antibodies, and mouse anti-Fibrillarin (Sigma-Aldrich), anti-β-Galactosidase (MP Biomedicals), anti-HA (Santa Cruz Biotechnology), anti-Osa and anti-Broad (Hybridoma Bank) antibodies. We also used rabbit anti-Dib (Parvy et al., 2005), and guinea pig anti-Neverland (Ohhara et al., 2017), anti-Spok (Gibbens et al., 2011), anti-Phtm and anti-Shroud (Shimada-Niwa and Niwa, 2014) antibodies. All these antibodies were a gift from Michael O'Connor and Ryosuke Niwa (University of Tsukaba, Japan). Alexa Fluor secondary antibodies (used at 1:200 dilution) were from Invitrogen. The nuclei were stained with DAPI (Invitrogen). Imaginal wing discs were dissected, fixed and stained as described previously (de Celis, 1997). In all immunostainings shown in Figs 5 and 8 wing discs were taken from larvae obtained after 24 h egg laying periods from parents of the appropriate genotypes, and the larvae were dissected 7 days after egg laying (AEL). Confocal images were taken using a LSM750 confocal microscope (Zeiss). All images were processed with the program ImageJ 1.45 s (NIH) and Adobe Photoshop 24.7.0.

### Transmission electron microscopy

Third instar larvae (*phtm-Gal4 UAS-GFP/UAS-GFP* and *tub-Gal80^ts^/UAS-salm-i; phtm-Gal4/UAS-salr-i*) grown for 2 days at 25°C, for 5 days at 17°C and for 5 days at 29°C were dissected and fixed in a formaldehyde/glutaraldehyde solution (4%/0.04%) for 2 h at room temperature and kept in the fixing solution for 2 days at 4°C. The prothoracic glands were separated from the larval carcasses and placed in epoxy resin, and all further processing was carried out in the transmission electron microscopy (TEM) facility at the CBMSO by Milagros Guerra. The samples were sectioned with an ultramicrotome and observed in a Transmission JEM1010 (Jeol) electro-microscope at 80 kV equipped with a TemCam F416 (TVIPS) using the software Emenu.

### Ecdysone treatments and survival assays

To synchronize larvae for ecdysone treatments, flies from appropriate genetic combinations were allowed to lay eggs for 3-4 h on grape-juice agar plates supplemented with yeast paste. First instar larvae were transferred to normal food for a 5 days period. Six third instar larvae were subsequently transferred (120 h AEL) to a 1.5 ml eppendorf tube containing 50 mg *Drosophila* instant food medium (Formula 4-24, Carolina), 10 mg of live yeast and 0.5-1 mg of 20-hydroxiecdysone in 200 µl of water. The number of pupae were counted during the following 4-day period and the rescue was expressed as the average ratio in different replicates between pupae and total number of larvae.

### Statistical analysis and graphical representation

All numerical data, including wing size and cell size values, were collected, processed and represented graphically in Microsoft Excel or GraphPad Prism version 8.0.1 for Windows. *P*-values were grouped in values of statistical confidence of 95% (*P<0.05), 99% (**P<0.01) and 99.9% (***P<0.001).

## Supplementary Material



10.1242/develop.202751_sup1 Supplementary information
