# Supplementary material for: Developmental analysis of Spalt function in the Drosophila prothoracic gland
Source: Development. 2024 Aug 27;151(16):dev202751. doi: 10.1242/dev.202751 (PMC11385645; doi:10.1242/dev.202751)
Supplement: Supplementary information [file develop-151-202751-s1.pdf]

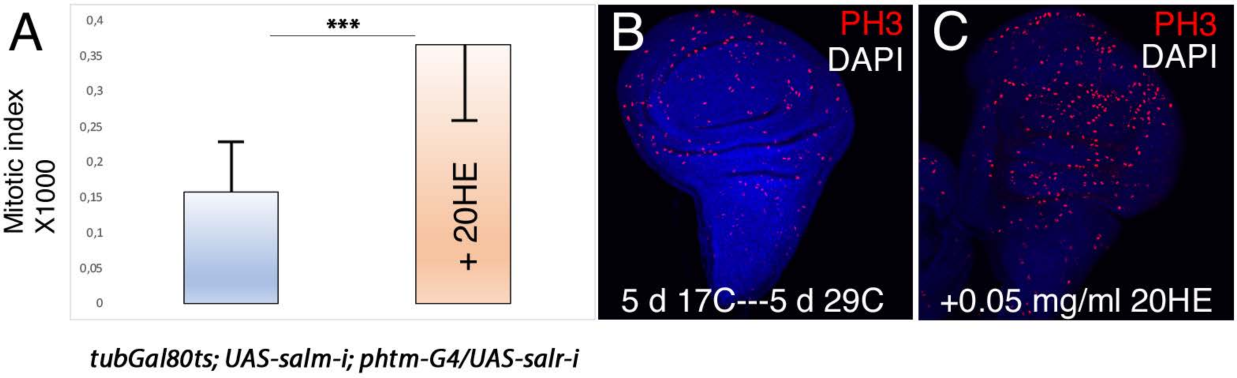

Fig. S1. Rescue of cell proliferation in the wing imaginal disc by 20HE in sal mutant larvae
(A) Mitotic index (number of cells in mitosis / wing blade area) *in tubGal80^{ts}/+; UAS-salm-RNAi/+; phtm-Gal4 UAS-GFP/UAS-salr-RNAi* grown 2 days at 25 °C, 5 d at 17 °C and 5 d at 29 °C raised in normal media (blue) or 24 h in media supplied with 0.05 mg/ml of 20HE (red). (B-C) Representative examples of wing discs in mutant larvae grown without 20HE (B) or supplied 24 h before dissection with 0.05 mg/ml of 20HE (C). The expression of pH3 is in red, and the expression of DAPI in blue.

Development • Supplementary information

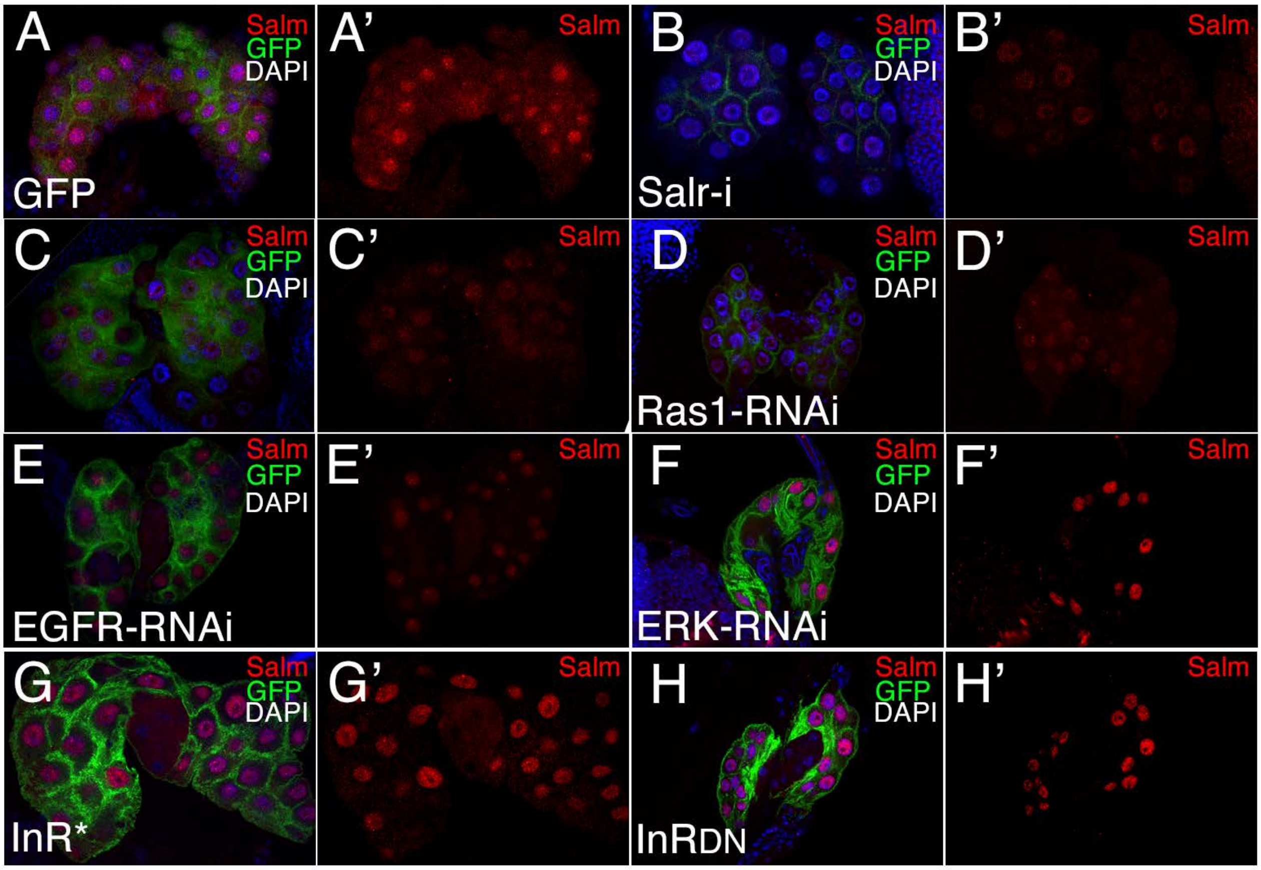

Fig. S2. Expression of Salm in the PG is not affected by changes in Ras/ERK or InR activity

(A-B) Expression of Salm (red) in control (*phtm-Gal4 UAS-GFP/+*; A) and *salr* knockdown (*phtm-Gal4 UAS-GFP/UAS-salr-RNAi*; B) PG. (C-H) Expression of Salm (red) after genetic modifications in components of the Ras/ERK and InR pathways: overexpression of Ras1^{V12} (*phtm-Gal4 UAS-GFP/UAS-Ras1*^{V12}; C-C'), loss of *Ras1* (*phtm-Gal4 UAS-GFP/UAS-Ras1-RNAi* ; D-D'), loss of *EGFR* (*phtm-Gal4 UAS-GFP/UAS-EGFR-RNAi*; E-E'), loss of *ERK* (*phtm-Gal4 UAS-GFP/UAS-rolled-RNAi*; F-F'), overexpression of constitutively activated InR (*phtm-Gal4 UAS-GFP/UAS-InR**; G-G') and overexpression of a dominant negative form of InR (*phtm-Gal4 UAS-GFP/UAS-InR*^{DN}; H-H'). The expression of GFP is in green, and the expression of DAPI in blue. All PC were from 7 d AEL larvae.

Development • Supplementary information

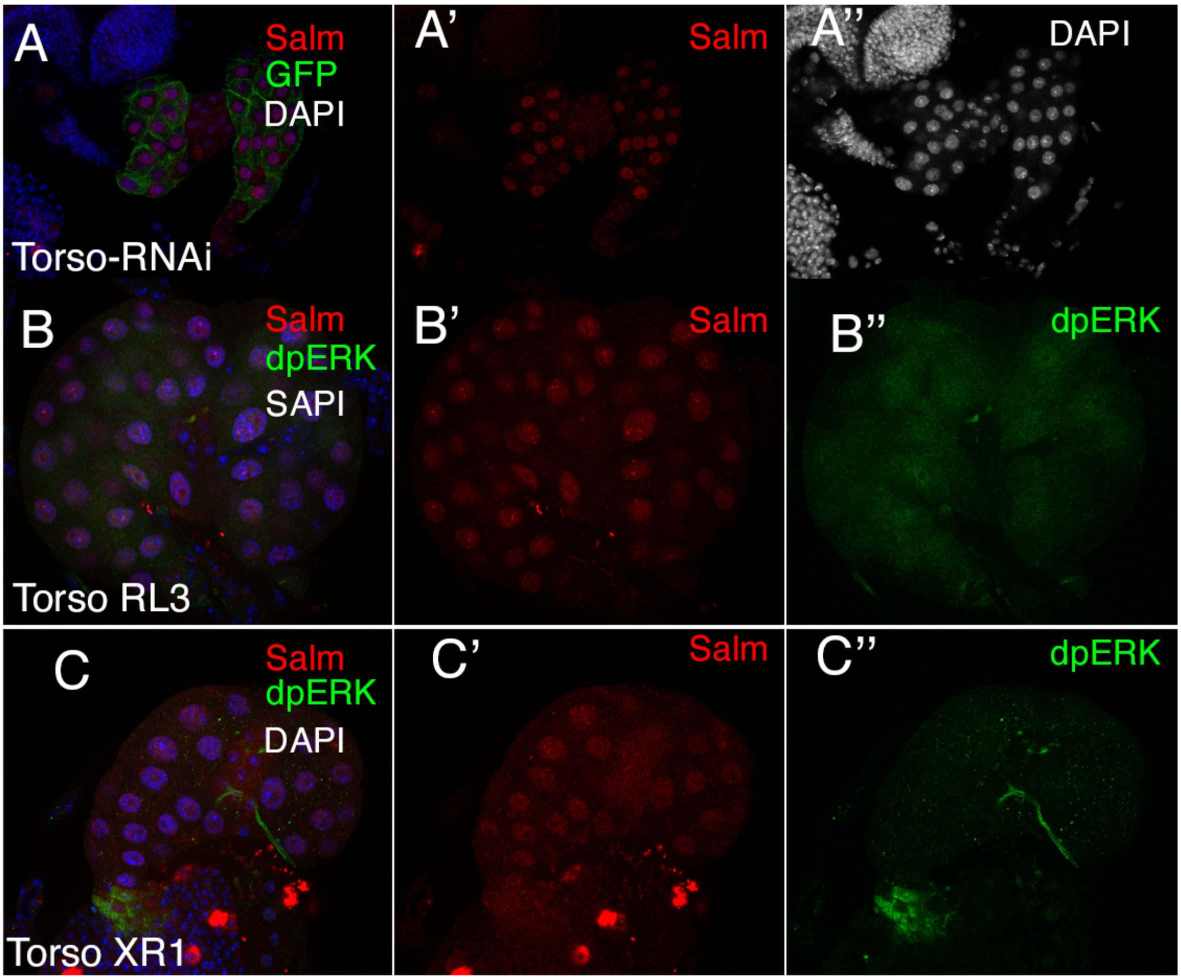

Fig. S3. Expression of Salm and dpERK in the PG after modifications or Torso activity

(A) Expression of Salm (red) in *torso* knockdown PG (*phtm-Gal4 UAS-GFP/UAS-torso-RNAi*; Torso-RNAi). (B) Expression of Salm (red) and dpERK (green) in the *torso* gain-of-function allele RL3 (tor^{RL3}). (C) Expression of Salm (red) and dpERK (green) in the *torso* loss of function allele XR1 (tor^{XR1}). The expression of GFP is in green in A, and DAPI is in blue. Individual red and green channels are shown in A'-C' and A''-C'', respectively.

Development • Supplementary information

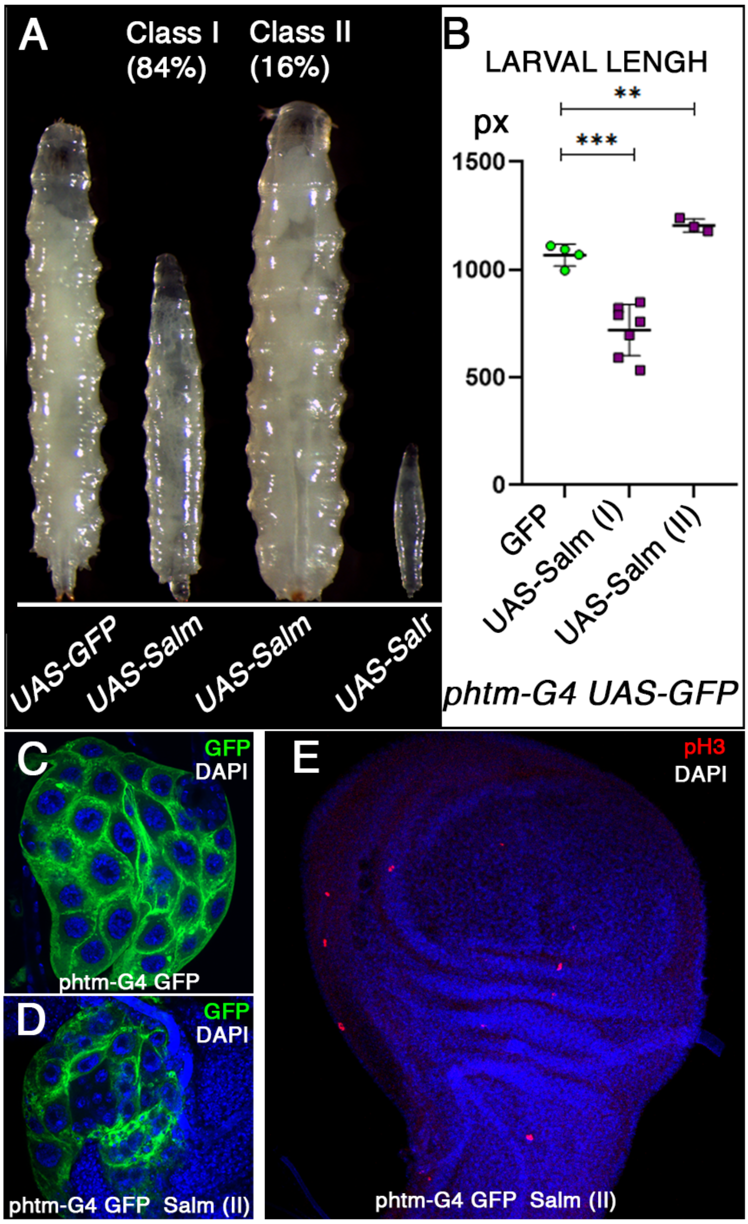

Fig. S4. Dominant negative effects of Salm and Salr overexpression in the PG

(A) Larval phenotypes obtained after overexpression of *salm* (Class I and Class II, *phtm-Gal4 UAS-GFP/UAS-salm*; *UAS-Salm*), *salr* (*phtm-Gal4 UAS-GFP/UAS-salr*; *UAS-Salr*) and control larvae (*pthm-Gal4 UAS-GFP/+*; GFP). (B) Quantification of larval length of class I and class II *phtm-Gal4 UAS-GFP/UAS-salm* larvae. (C-D) PG of control (*pthm-Gal4 UAS-GFP/+*, C) and class II *phtm-Gal4 UAS-GFP/UAS-salm* larvae. (E) Cell proliferation (phospho-Histone 3, red) in later third instar wing disc of class II *phtm-Gal4 UAS-GFP/UAS-salm* larvae.

Development • Supplementary information

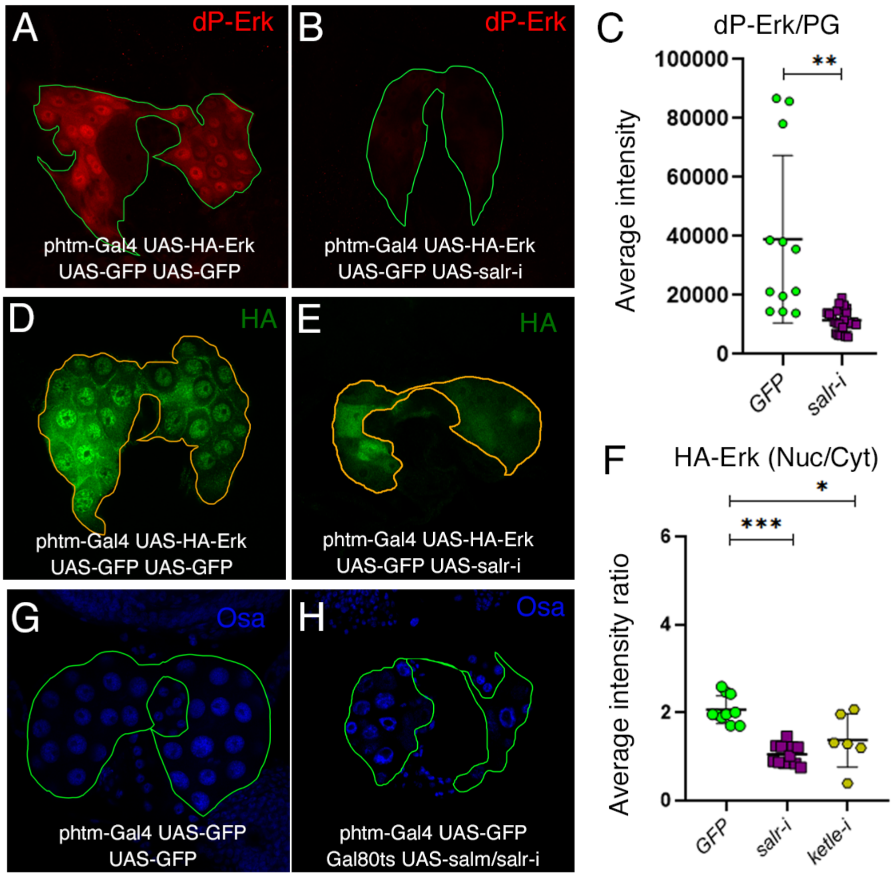

Fig. S5. Effects of *salr* knockdown on ERK activation and nuclear localization

(A-B) Expression of dP-ERK (red) in the PG of control larvae over-expressing HA-ERK (*UAS-HA-ERK/UAS-GFP*; *phtm-Gal4 UAS-GFP/+;* A) and in *salr* knockdown (*UAS-HA-ERK/+; phtm-Gal4 UAS-GFP/UAS-salr-RNAi*; B). (C) Quantification of dP-ERK intensity levels measured in individual PGs (C) extracted from *UAS-HA-ERK/UAS-GFP*; *phtm-Gal4 UAS-GFP/+* (GFP) and *UAS-HA-ERK/+; phtm-Gal4 UAS-GFP/UAS-salr-RNAi* (salr-i). (D-E) Expression of HA-ERK (HA; green) in the PG of *UAS-HA-ERK/UAS-GFP*; *phtm-Gal4 UAS-GFP/+* (D) and *UAS-HA-ERK/+; phtm-Gal4 UAS-GFP/UAS-salr-RNAi* (E) larvae. (G-H) Expression of Osa (Osa; blue) in the PG of *phtm-Gal4 UAS-GFP/UAS-GFP* (G) and *UAS-salm-RNAi/tubGal80ts; phtm-Gal4/UAS-salr-RNAi* (H). (F) Quantification of nucleus-cytoplasmic ratio of HA-ERK in the PG of *UAS-HA-ERK/UAS-GFP*; *phtm-Gal4 UAS-GFP/+* (GFP), *UAS-HA-ERK/+*; *phtm-Gal4 UAS-GFP/UAS-salr-i* (salr-i) and *UAS-HA-ERK/+*; *phtm-Gal4 UAS-GFP/UAS-Fs(2)Ket-RNAi* (ketel-i).
