# Supplementary material for: Developmental analysis of Spalt function in the Drosophila prothoracic gland
Source: Development. 2024 Aug 27;151(16):dev202751. doi: 10.1242/dev.202751 (PMC11385645; doi:10.1242/dev.202751)
Supplement: Supplementary file 1 [file develop-151-202751_review_history.pdf]

Developmental analysis of Spalt function in the *Drosophila* prothoracic gland

Cristina M. Ostalé, Diego Pulido, Patricia Vega-Cuesta, Ana López-Varea and Jose F. de Celis

DOI: 10.1242/dev.202751

Editor: Cassandra Extavour

Review timeline

Original submission:	31 January 2024
Editorial decision:	12 March 2024
First revision received:	10 June 2024
Editorial decision:	1 July 2024
Second revision received:	8 July 2024
Accepted:	17 July 2024

Original submission

First decision letter

MS ID#: DEVELOP/2024/202751

MS TITLE: Developmental analysis of Spalt function in the Drosophila ring gland

AUTHORS: Jose F. de Celis, Diego Pulido, Cristina M Ostale, and Ana Lopez-Varea

I have now received all the referees' reports on the above manuscript, and have reached a decision. The referees' comments are appended below, or you can access them online: please go to BenchPress and click on the 'Manuscripts with Decisions' queue in the Author Area.

As you will see, the referees express considerable interest in your work, but have some significant criticisms and recommend a substantial revision of your manuscript before we can consider publication. If you are able to revise the manuscript along the lines suggested, which may involve further experiments, I will be happy receive a revised version of the manuscript. Your revised paper will be re-reviewed by one or more of the original referees, and acceptance of your manuscript will depend on your addressing satisfactorily the reviewers' major concerns. Please also note that Development will normally permit only one round of major revision. If it would be helpful, you are welcome to contact us to discuss your revision in greater detail. Please send us a point-by-point response indicating your plans for addressing the referees' comments, and we will look over this and provide further guidance.

Please especially consider the comments of Reviewer 1, who I believe makes important points regarding the depth of understanding of the mechanisms underlying the phenotypes that are beautifully presented here. Strengthening this aspect of the MS will be important to consider if you choose to revise and resubmit.

Please attend to all of the reviewers' comments and ensure that you clearly highlight all changes made in the revised manuscript. Please avoid using 'Tracked changes' in Word files as these are lost in PDF conversion. I should be grateful if you would also provide a point-by-point response detailing how you have dealt with the points raised by the reviewers in the 'Response to Reviewers' box. If you do not agree with any of their criticisms or suggestions please explain clearly why this is so.

© 2024. Published by The Company of Biologists under the terms of the Creative Commons Attribution License (https://creativecommons.org/licenses/by/4.0/).

Reviewer 1

Advance summary and potential significance to field

The manuscript by Ostalé and coauthors explores the function of Spalt proteins salr and salm, on prothoracic gland development and function. Here, the authors show that knock down of salr and salm in the prothoracic glands of larvae causes phenotypes that resemble the hallmarks of ecdysone deficiency including delayed pupariation, reduced cell divisions in the wing disc, and reduced expression of at least one Halloween gene. The authors then explore the potential cause of these effects and find that knocking down salr and salm affects the distribution of DNA, the size of the nucleolus, and the shape of the nuclear membrane.
Further, they find that ERK signalling is reduced when salr is knocked down in the prothoracic gland.

I think this presents an interesting story and the data is of high quality. I think one of the novelties of this paper is that it uncovers a clear link between nuclear structure and the ability of the prothoracic glands to perform their function, to produce ecdysone. The authors make a reasonable effort to quantify their findings, where possible, which is greatly appreciated.

Comments for the author

In the introduction and overall justification of the study system, it's not very clear why the authors chose to study Spalt proteins in the prothoracic gland cells. These cells are excellent for studies of nuclear structure, because they are polyploid and so the nuclei are large. Also, the phenotypes associated with disrupting prothoracic gland function are really well understood. I wonder if more of this justification could be woven into the introduction to make the motivation behind the study crystal clear. As the story is currently setup, it reads like you expect these proteins to be involved in the regulation of ecdysone.

The results text that discusses Figure 1C say that 20% of phtm > salri larvae make it to pupariation, but this isn't reflected in this graph. I expect that this is because it takes them longer than 22 days to pupariate? So that your data align with the text, it would be work presenting a separate plot that shows the % of larvae that make it to each stage for each manipulation.

While in the methods you describe how you stage larvae for the 20E rescue and survival experiments, some description of how you stage early and late wing discs in Figure 3 would be useful.

Similarly, for the data in Figures 4 and 5 it would be useful how the larvae were staged. Because ecdysone signalling pathway genes fluctuate over developmental time, it's useful to ensure that tissue is collected at the same developmental stage (e.g. wandering) to rule out differences caused by developmental progression.

Your findings that ERK localization was impacted, but overexpression of ERK did not rescue the phenotype. This aligns with the statements in the discussion that Salm/Salr proteins are important for nuclear structure and function and are unlikely to be directly regulating ecdysone production. However, in the results I found the switch to ERK signalling a bit surprising as it didn't seem to jive with the previous section on nuclear structure. I wonder if it would be worth reordering some of the data to present the ERK data first, then after presenting the ERK overexpression results suggest that maybe Spalt proteins are really affecting nuclear structure. This might make the story flow a bit better.

Minor comments:

- In figure 1C, it would be helpful to label your x axis (days?). Also, the y axis is labelled phm-G4 but you use the phtm abbreviation in the text and other parts of the figure. I would modify for clarity.

© 2024. Published by The Company of Biologists under the terms of the Creative Commons Attribution License (https://creativecommons.org/licenses/by/4.0/).

- Is it possible that the smaller than normal P0206-GAL4 > salm i, salr i pupae were actually L2 pupae? This has been described in other studies reducing ring gland size.
- For typos, please see edited pdf.

Reviewer 2

Advance summary and potential significance to field

In the manuscript, Ostale et al. investigate the role of Spalt transcription factors in the development and function of the prothoracic gland (PG) in the fruit fly Drosophila melanogaster. The study shows that the Spalt proteins Salm and Salr are required for ecdysone synthesis in the PG and are particularly important for regulating the expression of phantom (phtm), which encodes one of the key ecdysone biosynthetic enzymes.
Furthermore, the manuscript shows that loss of spalt function in the PGs results in abnormalities in nuclear organization, including chromatin distribution, nucleolus size, and nuclear envelope morphology. These abnormalities are associated with impaired ERK signaling, likely due to perturbations in nuclear architecture that affect nuclear pore function.
Over the past decade, transcriptional regulatory networks controlled by multiple regulators of transcription, chromatin remodeling, and endoreplication have been shown to be essential for the spatiotemporal expression control of Halloween genes in Drosophila melanogaster. However, it is still unclear how each of the Halloween genes is transcriptionally regulated in a spatiotemporally specific manner. In this context, the most interesting finding of this manuscript for the reviewer is that Spalt may be specifically involved in the regulation of phtm expression. In addition, the study also elucidates the role of Spalt in coordinating nuclear organization within the PG. This finding potentially sheds light on the role of intracellular architecture in ecdysone biosynthesis, which is something new in this field of research.

Comments for the author

Although the authors report several interesting observations in this manuscript, this reviewer cannot strongly recommend publication of the manuscript in Development. The main reason for this is that most of what the authors report in this manuscript is rather descriptive, and the authors do not provide mechanistic insights into how Spalt transcription factors affect the expression of phtm, the phosphorylation of Erk, and ultimately the nuclear envelope and nucleolus. Although the reviewer understands the importance of descriptive research in science, the reviewer does not believe that this paper meets the standard level of Development because the manuscript does not advance the understanding of detailed mechanisms.

[Major concerns]
(1) It is interesting that Spalt is involved in ERK phosphorylation in the PG. The reviewer expects that the authors will investigate how Spalt is involved in Erk-mediated signaling pathways in PG, such as the PTTH-Torso pathway, the Jelly Belly-Alk pathway, and the Pvf-Pvr pathway. In particular, it is very unfortunate that the relationship with Spalt and the PTTH-Torso pathway has not been investigated in the manuscript. Several potential experiments are in the reviewer's mind. For example, can the precocious phenotype caused by RasV12 overexpression (reported in Rewitz et al. Science 2009) be suppressed by RNAi of Salm or Salr? Can the developmental delay of PTTH mutants and PG-specific Torso RNAi be rescued by overexpression of Salm or Salr? Do the expression and protein levels of Salm and Salr change in the PG of these loss-of-function animals?

(2) The authors find that Spalt is involved in regulating the expression of phtm, but not nvd and sro, and that the loss of Spalt function phenotype can be rescued by overexpression of phtm. The reviewers consider these findings to be the most important discovery by the authors. To maximize the value of this discovery, the reviewer strongly asks the authors to analyze the expression of all other Halloween genes, namely noppera-bo (nobo), spookier (spok), disembodied (dib), and shadow (sad), in the salm/salr RNAi PG. If it is confirmed that Spalt is indeed a transcription factor acting only on phtm, it will be recognized as a new transcription factor involved in the expression of the very specific subset of Halloween genes, such as ouija board, seance, and malting defective. This will be an important finding for the field of ecdysone biology.

© 2024. Published by The Company of Biologists under the terms of the Creative Commons Attribution License (https://creativecommons.org/licenses/by/4.0/).

(3) The mitotic phenotype of the imaginal disc described in Figure 3 is also interesting. The must investigate whether this mitotic phenotype can be reversed by feeding with 20E.

(4) The reviewer simply cannot understand the necessity of the data described in P15 and Fig.7. What is said in these data is not related in any way to the functionality of Spalt.

[Minor concerns]
(i) These days, the reviewer believes that the "data not shown" statement is basically inappropriate. In this manuscript, P9-L23, P10-L19, P12-L24, and P13-L12 have "data not shown". The reviewer believes that the manuscript should be revised to properly present these data as Supplementary Information.

(ii) P3, L17-18: "These proteins belong to the cytochrome P450 family of enzymes with monooxygenase activity": This sentence does not accurately describe the Halloween genes. Some Halloween genes, such as Nobo, Nvd, and Sro, do not encode P450 but other types of enzymes (GST, Rieske, and SDR, respectively). The authors need to revise this explanation.

(iii) P6: The authors must describe a source of UAS-nvd used in this study.

(iv) P10, L2-3: While the authors mention the knockdown efficiency for salm-RNAI somehow they don't mention the efficiency for salr-RNAI. The authors need to clarify this point.

(v) P10, L19-20 and Fig. 1D, G: Based on the description in the manuscript, it is unclear whether the authors performed a temperature shift experiment with tub-Gal80ts/UAS-salm-i; phtm-Gal4/UAS-salr-I to obtain the larval phenotype or not. Was the experimental condition the same as that described by the authors at P11, L18? Please clarify.

(vi) P11, L4: "Fig. 2A-F" should read "Fig. 2A-D, F". Alternatively, authors should switch between the current Fig. 2E and Fig. 2F.

(vii) P12-L12-13: Why did the authors use salr RNAi here instead of salm and salr double RNAi? The reviewer does not see any particular reason.

(viii) P12, L22-P13, L3 and Figs. 4M, 4N: The reviewer would like to see a graph showing the results of an analysis using a larger number of larvae to confirm what percentage of larvae are rescued into pupae by feeding on 20E.

(ix) P13, L12-16: It is better to include the immunostaining data of the anti-Phtm antibody along with Figures 5E-H. The rabbit anti-Phtm antibody has already been generated by Michael B. O'Connor, Minnesota, USA.

(x) P13, L11: "Nvn" should be corrected to "nvd" (italicized).

(xi) P14, second paragraph, and Fig. 6C-F: For the general reader, please indicate with arrows, arrowheads, or other markers which are DNA, nucleoli, and nuclear envelopes, and also clarify these points in the figure legend.

(xii) P17, L11: "Prothoracic gland" should be abbreviated to "PG".

(xiii) In almost all figures, the blue letters of "DAPI" are barely visible. The authors need to consider a better way to show them.

(xiv) Fig. 1A, 1B" On the figure, it is better to describe the genotypes used by the authors.

(xv) Figure 1F, 1G: On the figure, it is better to describe "phtm-Gal4" as the driver used.

(xvi) Fig. 2D-F: Can the authors show any counterstaining data to make sure that the PGs are present in these photographs?

(xvii) As listed below, authors should properly cite some references in the manuscript.

© 2024. Published by The Company of Biologists under the terms of the Creative Commons Attribution License (https://creativecommons.org/licenses/by/4.0/).

P4, L3-5: The authors should also mention Pvf and Jelly Belly, and also cite Pan and O'Connor, Cell Reports 2021.

P4, L10, and P12-L21-22: Instead of Niwa & Niwa 2016, authors should cite Komura-Kawa et al., PLOS Genetics 2015.

P5, L17: In addition to Danielsen et al. 2014, the following article should also be cited as this paper also focuses on ventral vein absence/drifter.
Yunpo Zhao, Bo Gustav Lindberg, Shiva Seyedoleslami Esfahani, Xiongzhuo Tang, Stefano Piazza, Ylva Engström (2021) Stop codon readthrough alters the activity of a POU/Oct transcription factor during Drosophila development. BMC Biol 19(1):185. doi: 10.1186/s12915-021-01106-0.

P12-L12, In addition to Shirai et al. 2012, the following article should also be cited.
Christian F Christensen, Takashi Koyama, Stanislav Nagy, E Thomas Danielsen, Michael J Texada, Kenneth A Halberg, Kim Rewitz (2020) Ecdysone-dependent feedback regulation of prothoracicotropic hormone controls the timing of developmental maturation.
Development 147(14):dev188110. doi: 10.1242/dev.188110.

P14-L5: The reviewer believes that "including Kni and Vvl (Danielsen et al. 2014)" should be deleted. Instead, it is better to cite Kamiyama & Niwa Frontiers in Physiology 2022 right after "other transcription factors".

First revision

Author response to reviewers' comments

We would like to thank both reviewers for their comments, which have push us to produce what we think is a much better version of the manuscript. In what follows we explain what we have done to answer all reviewer’s criticism and suggestion.

Reviewer 1

Comments for the Author:

1) In the introduction and overall justification of the study system, it’s not very clear why the authors chose to study Spalt proteins in the prothoracic gland cells. These cells are excellent for studies of nuclear structure, because they are polyploid and so the nuclei are large. Also, the phenotypes associated with disrupting prothoracic gland function are really well understood. I wonder if more of this justification could be woven into the introduction to make the motivation behind the study crystal clear. As the story is currently setup, it reads like you expect these proteins to be involved in the regulation of ecdysone.

-We think this is a very valuable suggestion and have inserted in the introduction the following paragraph:

Page 4, Inserted: “*The cells in the PG are polyploid, displaying during larval life a cell cycle in which the S phase is followed with G phases without mitosis, reaching a DNA content of 64C (Ohhara et al., 2017). Polyploidy poises challenges to chromosomal stability, gene expression regulation and nuclear envelope function, and because of their large nuclear size and resistance to apoptotic signals, the PG constitutes a convenient system to analyze the interrelationships between gene expression and nuclear chromatin organization. We have used this experimental setting to better understand the function of the Spalt proteins, two C2H2 transcriptional regulators which function had only been previously characterized in diploid cells.*”
2) The results text that discusses Figure 1C say that 20% of phtm > salri larvae make it to pupariation, but this isn’t reflected in this graph. I expect that this is because it takes them longer

© 2024. Published by The Company of Biologists under the terms of the Creative Commons Attribution License (https://creativecommons.org/licenses/by/4.0/).

than 22 days to pupariate? So that your data align with the text, it would be work presenting a separate plot that shows the % of larvae that make it to each stage for each manipulation.

We understand the reviewer point, because we didn´t make a good job of explaining the phtm>salri phenotype in all its details. In this manner, Fig. 1C was only a simplification of the results that we agree leads to confusion. We have substituted this figure with alternative panels (new Fig. 1C-D) that encapsulates the different phenotypes in a more quantitative and comprehensible manner.

We modified the following paragraph:

Page 10-11, deleted: “Larvae in which only the expression of *salm* is knocked-down (*phtm-Gal4 UAS- GFP/UAS-salm-i*) show a weak phenotype consisting in a two days extension of larval development and a total delay of 3.5 days in adult eclosion (Fig. 1C). When only the expression of *salr* is knockdown (*phtm- Gal4 UAS-GFP; UAS-salr-i/+*), the phenotype is stronger, and 80% of mutant larvae display a developmental arrest in late L3 (Fig. 1C). The remaining 20% of phtm-Gal4 UAS-GFP/UAS-salr-i larvae can progress with a delay to pupariation, but the resulting pupae die in early stages of pupal development (Fig. 1C). Larvae in which both *salm* and *salr* expression are knocked down (*phtm-Gal4 UAS-GFP/UAS-salm-i; UAS-salr-i/+*) show a fully penetrant developmental arrest as early L3 larvae (Fig. 1C).”

Page 10-11 inserted: “Larvae in which only the expression of *salm* is knocked-down (*phtm-Gal4 UAS- GFP/UAS-salm-i*) progress with normal frequencies throughout the larval stages and pupal development (Fig. 1C), and only show a weak delay in pupariation (Fig. 1C-D). When only the expression of *salr* is knockdown (*phtm-Gal4 UAS-GFP; UAS-salr-i/+*), the phenotype is stronger, and only 24% of mutant larvae enter pupariation in the first 9 days AEL (Fig. 1D). This number increases to 43% after 15 days AEL (Fig. 1D), and the remaining *phtm-Gal4 UAS-GFP/UAS-salr-i* larvae display a developmental arrest in late L3 (Fig. 1D). Most *phtm-Gal4 UAS-GFP/UAS-salr-i* pupae die during pupal development, with a final rate of adult eclosion of 5% (Fig. 1D). Larvae in which both *salm* and *salr* expression are knocked down (*phtm-Gal4 UAS-GFP/UAS-salm-i; UAS-salr-i/+*) show larval lethality in L1 and L2 (Fig. 1D), and the 30% of surviving L2 larvae display a fully penetrant developmental arrest as late L2 larvae (Fig. 1D, G-I).”

3) While in the methods you describe how you stage larvae for the 20E rescue and survival experiments, some description of how you stage early and late wing discs in Figure 3 would be useful.

-We added the following sentence:

Page 7, added: “Wing discs were taken from early L3 (EL3; 90 +/- 12 hours AEL) and late L3 (LL3; 120 +/- 12 h AEL) larvae of *phtm-Gal4 UAS-GFP/UAS-GFP*, from late L2 *UAS-salm-RNAi/+; phtm-Gal4 UAS-*

GFP/UAS-salr-RNAi (90 +/- 12 hours AEL) and from late L3 *tubGal80^{ts}/+; UAS-salm-RNAi/+; phtm-Gal4 UAS-GFP/UAS-salr-RNAi* larvae grown 2 days at 25 C, 5 days at 17 and 5 days at 29 C.

4) Similarly, for the data in Figures 4 and 5 it would be useful how the larvae were staged. Because ecdysone signaling pathway genes fluctuate over developmental time, it’s useful to ensure that tissue is collected at the same developmental stage (e.g. wandering) to rule out differences caused by developmental progression.

-We added the following sentence:
Page 8-9, added: “In all immunostainings shown in Figures 5 and 8 wing discs were taken from larvae obtained after 24 h egg laying periods from parents of the appropriate genotypes, and the larvae were dissected 7 days after egg laying (AEL).”

We also added to the corresponding figure legends and material and methods the developmental age of the larvae used for each experiment.

5) Your findings that ERK localization was impacted, but overexpression of ERK did not rescue the phenotype. This aligns with the statements in the discussion that Salm/Salr proteins are important

© 2024. Published by The Company of Biologists under the terms of the Creative Commons Attribution License (https://creativecommons.org/licenses/by/4.0/).

for nuclear structure and function and are unlikely to be directly regulating ecdysone production. However, in the results I found the switch to ERK signaling a bit surprising as it didn't seem to jive with the previous section on nuclear structure. I wonder if it would be worth reordering some of the data to present the ERK data first, then after presenting the ERK overexpression results suggest that maybe Spalt proteins are really affecting nuclear structure. This might make the story flow a bit better.

-We agree that there is a jump from Sal and the nuclear envelope to the relationships between Sal genes and ERK signaling. For this reason, we have taken your suggestion to introduce a new paragraph to explain properly this transition. We think the ERK overexpression result is not really informative, because ERK overexpression perse does not affect pupariation time nor rescues the *sal* mutant phenotype. We have now introduced novel data concerning the relationships between Ras/ERK and Sal functions that we think should help to clarify this final section of the manuscript. The changes we have made to the manuscript are:

-**Page 17, inserted**: "*Considering the effects of salm/salr knockdown on nuclear morphology, and the similarity of the salm/salr phenotype with loss of emb or Fs(2)Ket, and with Lamin overexpression, it is possible that in salm/salr loss of function conditions the import of activated ERK into the nucleus was compromised.*
-**Page 16-17, deleted**: "We were only able to visualize dP-ERK in the PG when ERK is over-expressed (UAS-HA-Erk/+; phtm-Gal4 UAS-GFP/+; Fig. 8A)."
We change the fixation protocol and were now able to visualize dP-ERK without the need to overexpressed ERK. This allowed us to make a new set of experiments describing the interactions between ERK activation and Sal function, and these has led to the following changes:

-Fig. 8 is now Supplementary Fig. 3.

-Added a new Fig. 8, Fig. 9 and Supple Fig. 2, 3 and 5 to describe Ras/Salr interactions

-**Page 17-18, inserted:** "*The expression of dP-ERK is detected in the PG of late third instar larvae (Ohhara et al., 2015; Cruz et al., 2020), where dP-ERK accumulates in both the nucleus and cytoplasm (Fig. 8A-B, R). Consistently with previous observations (Cruz et al., 2020), we find that dP-ERK expression is very much reduced after knockdowns of Ras1 (Fig. 8C-D, Q), and knockdowns of the receptors EGFR (Fig. 8 G-H, S) and Torso (Fig. 8I-J, S). Similarly, gain- and loss-of-function alleles of torso have opposite effects on dP- ERK accumulation (Fig. 8K-L). The levels of dP-ERK increase when an activated version of Ras1 ($Ras1^{V12}$) is expressed in the PC (Fig. 8 E-F, Q). These changes of dP-ERK levels of expression are accompanied by a slight reduction in the nucleus-cytoplasm ration of dP-ERK accumulation (Fig. 8R), and in no case the expression of Salm is affected in PG glands with alterations in Ras/ERK signaling (Supple. Fig. 1-2). Surprisingly, the knockdown of salr in the PC leads to a strong reduction in dP-ERK expression levels (Fig. 8M-L, Q), and in this case the nucleus-cytoplasm ration of dP-ERK accumulation is very much reduced (Fig. 8R). To better define the impact of sal function on ERK activation we carry out a rescue experiment in a salr knockdown background using the expression of $Ras1^{V12}$. We found that $Ras1^{V12}$ can to a large extent rescue the loss of dP-ERK caused by loss of salr (Fig. 8O-P, Q-R). This rescue of dP-ERK levels is accompanied by a phenotypic rescue of pupariation time and in the frequency of pupae (Fig. 9). In fact, the frequency of mutant pupae, as well as the pupariation time of salr-i/$Ras1^{V12}$ larvae is very similar to that of $Ras1^{V12}$ ones (Fig. 9E-F). Taken together, these results suggest that reduced ERK activation is a fundamental aspect of the sal loss of function phenotype in the PC. We tried to make the converse experiments, rescue of Ras/ERK signaling by Salm/Salr overexpression. However, overexpression of Salm or Salr in the PG results in phenotypes which are reminiscent of loss of salm/salr function (Supple. Fig. 3), which we think are caused by a dominant negative effect of these proteins when over-expressed. These phenotypes include the formation of small larvae (84%) and large L3 larvae with extended L3 development (16%). Only 17% of these large L3 larvae can enter pupariation, dying as early pupae. The corresponding PG of the LL3 larvae appear very much disorganized and with cells of different sizes (Supple. Fig. 3).*".

-**Page 17**, changed the paragraph structure of the ERKHA data, which is now illustrated in Supple. Fig. 4.

© 2024. Published by The Company of Biologists under the terms of the Creative Commons Attribution License (https://creativecommons.org/licenses/by/4.0/).

Added: "*To better visualize ERK nuclear accumulation in wild type and sal mutant conditions, we expressed a tagged ERK protein (HA-ERK) in the PC.*"

Deleted: "indicating that the normal rise in ERK phosphorylation that takes place at the end of larval development fails in cells with reduced salr expression"

Minor comments:
- In figure 1C, it would be helpful to label your x axis (days?). Also, the y axis is labelled phm-G4 but you use the phtm abbreviation in the text and other parts of the figure. I would modify for clarity.

Fig. 1C has been substituted by two new panels (Fig. 1C and Fig. 1D) to reflect the variability of the phenotypes in a quantitative manner.

- Is it possible that the smaller than normal P0206-GAL4 > salm i, salr i pupae were actually L2 pupae? This has been described in other studies reducing ring gland size.

We think that the *P0206-GAL4 > salm i, salr-i* pupae correspond to L3 pupae, as they have the characteristic L3 mouth hooks. The reviewer suggestion led us to re-evaluate the smaller than normal *phm-Gal4/salmi/salri* larvae, which we interpreted as smaller than normal L3. By looking at their mouth hooks we can now conclude that these larvae are actually L2 larvae, and we have consequently changed our previous statement. We also added new panels to Fig. 1 to illustrate the mouth hooks of L2 wild type and *phm- Gal4/salmi/salri*.

Page 11, added: "*Larvae in which both salm and salr expression are knocked down (phtm-Gal4 UAS- GFP/UAS-salm-i; UAS-salr-i/+) show larval lethality in L1 and L2 (Fig. 1D), and the 30% of surviving L2 larvae display a fully penetrant developmental arrest as late L2 larvae (Fig. 1D, G-I).*"
- For typos, please see edited pdf.

-We made all the suggested corrections to the text

Reviewer 2
Comments for the Author:

1) Although the authors report several interesting observations in this manuscript, this reviewer cannot strongly recommend publication of the manuscript in Development. The main reason for this is that most of what the authors report in this manuscript is rather descriptive, and the authors do not provide mechanistic insights into how Spalt transcription factors affect the expression of phtm, the phosphorylation of Erk, and ultimately the nuclear envelope and nucleolus. Although the reviewer understands the importance of descriptive research in science, the reviewer does not believe that this paper meets the standard level of Development because the manuscript does not advance the understanding of detailed mechanisms.

-We respect the reviewer statements, but have to disagree in one fundamental point. In this manuscript we identify a novel plausible mechanism by which a transcription factor affects gene expression without direct regulation of target genes. We agree that we cannot provide at this point a detailed molecular mechanism linking the function of Spalt as a heterochromatic DNA binding protein with its effects on nuclear envelope, nucleolus size, nuclear import of ERK and ultimately the expression of Halloween genes, but we believe these findings open new possibilities to understand a novel mode of gene regulation operating in the prothoracic gland, and this may have broader implications for the role of Spalt proteins and likely other transcriptional regulators.

2) It is interesting that Spalt is involved in ERK phosphorylation in the PG. The reviewer expects that the authors will investigate how Spalt is involved in Erk-mediated signaling pathways in PG, such as the PTTH- Torso pathway, the Jelly Belly-Alk pathway, and the Pvf- Pvr pathway. In particular, it is very unfortunate that the relationship with Spalt and the PTTH-Torso pathway has not been investigated in the manuscript. Several potential experiments are in the reviewer's mind. For example, can the precocious phenotype caused by RasV12 overexpression (reported in Rewitz et al. Science 2009) be suppressed by RNAi of Salm or Salr? Can the developmental delay of PTTH

© 2024. Published by The Company of Biologists under the terms of the Creative Commons Attribution License (https://creativecommons.org/licenses/by/4.0/).

mutants and PG-specific Torso RNAi be rescued by overexpression of Salm or Salr? Do the expression and protein levels of Salm and Salr change in the PG of these loss-of- function animals?

-We strongly agree with the reviewer that the relation we find between Spalt and ERK phosphorylation in the PG is worth of a more in-depth analysis. In order to partially answer the reviewer criticism, we have made many changes to this section of the manuscript, including additional experiments. We have also incorporated a new author to the manuscript (Patricia Vega), who has carried out these new experiments.

-**Added**:
a) Analysis of Salm expression in the PG of larvae mutant for the loss and gain of function Torso alleles *Tor*^{XR1} and *Tor*^{RL3}, and for larvae expressing the constructs EGFR-RNAi, Ras1-RNAi, Ras1^{V12} and torso alleles. These experiments show that Salm expression is not affected by changes in the activity of the Ras/ERK pathway (new Suppl. Figs. 2 and 3).
b) New data concerning the over-expression of Salm and Salr (Supple. Fig. 4). We found that overexpression of Salm or Salr in the PG results in phenotypes which are reminiscent of loss of *salm/salr* function, which we think are caused by a dominant negative effect of these proteins when over-expressed. These phenotypes include the formation of small L3 larvae (84%) and large L3 larvae with extended L3 development (16%). Only 17% of these large L3 larvae can enter pupariation, dying as early pupae. The corresponding PG of the LL3 larvae appear very much disorganized and with cells of different sizes (Supple. Fig. 4). Due to this dominant negative effect of Salm overexpression we cannot make the rescue experiments suggested by the reviewer (*UAS-sal* combined with *PTTH* mutants or *Torso-RNAi*).

c) New descriptions of dP-ERK expression in mutants of the Ras/ERK pathway, salr-i, Ras1^{V12} and *salr- i/Ras1*^{V12} (new Figure 8). We were able to visualize dP-ERK expression by increasing the time of sample fixation. This allowed us to make novel observations, and describe the interactions between Ras1 activation and *sal* loss of function conditions. The results indicate that over-activation of the Ras/ERK pathway rescue to a large extent the *salr-RNAi* phenotype.
D) A new Figure 8 and 9 illustrating the interactions between ERK activation and Sal function.

-To better explain and reinforce the analysis of Sal function with ERK activation we have made the following changes:

1) Page 17-18, also included in response to reviewer 1:

Added: "*Considering the effects of salm/salr knockdown on nuclear morphology, and the similarity of the salm/salr phenotype with loss of emb or Fs(2)Ket, and with Lamin overexpression, it is possible that in salm/salr loss of function conditions the import of activated ERK into the nucleus was compromised. The expression of dP-ERK is detected in the PG of late third instar larvae (Ohhara et al., 2015; Cruz et al., 2020), where dP-ERK accumulates in both the nucleus and cytoplasm (Fig. 8A-B, R). Consistently with previous observations (Cruz et al., 2020), we find that dP-ERK expression is very much reduced after knockdowns of Ras1 (Fig. 8C-D, Q), and knockdowns of the receptors EGFR (Fig. 8 G-H, S) and Torso (Fig. 8I-J, S). Similarly, gain- and loss-of-function alleles of torso have opposite effects on dP-ERK accumulation (Fig. 8K- L). The levels of dP-ERK increase when an activated version of Ras1 (Ras1*^{V12}*) is expressed in the PC (Fig. 8 E-F, Q). These changes of dP-ERK levels of expression are accompanied by a slight reduction in the nucleus-cytoplasm ration of dP-ERK accumulation (Fig. 8R), and in no case the expression of Salm is affected in PG glands with alterations in Ras/ERK signaling (Supple. Fig. 2-3). Surprisingly, the knockdown of salr in the PC leads to a strong reduction in dP-ERK expression levels (Fig. 8M-L, Q), and in this case the nucleus-cytoplasm ration of dP-ERK accumulation is very much reduced (Fig. 8R). To better define the impact of sal function on ERK activation we carry out a rescue experiment in a salr knockdown background using the expression of Ras1*^{V12}*. We found that Ras1*^{V12} *can to a large extent rescue the loss of dP-ERK caused by loss of salr (Fig. 8O-P, Q-R). This rescue of dP-ERK levels is accompanied by a phenotypic rescue of pupariation time and in the frequency of pupae (Fig. 9). In fact, the frequency of mutant pupae, as well as the pupariation time of salr-i/Ras1*^{V12} *larvae is very similar to that of Ras1*^{V12} *ones (Fig. 9E-F). Taken together, these results suggest that reduced ERK activation is a fundamental aspect of the sal loss of function phenotype in the PC. We tried to make the converse experiments, rescue of Ras/ERK signaling by Salm/Salr overexpression. However, overexpression of Salm or Salr in the PG results in phenotypes which*

© 2024. Published by The Company of Biologists under the terms of the Creative Commons Attribution License (https://creativecommons.org/licenses/by/4.0/).

are reminiscent of loss of salm/salr function (Supple. Fig. 4), which we think are caused by a dominant negative effect of these proteins when over-expressed. These phenotypes include the formation of small larvae (84%) and large L3 larvae with extended L3 development (16%). Only 17% of these large L3 larvae can enter pupariation, dying as early pupae. The corresponding PG of the LL3 larvae appear very much disorganized and with cells of different sizes (Supple. Fig. 4).”

(2) The authors find that Spalt is involved in regulating the expression of phtm, but not nvd and sro, and that the loss of Spalt function phenotype can be rescued by overexpression of phtm. The reviewers consider these findings to be the most important discovery by the authors. To maximize the value of this discovery, the reviewer strongly asks the authors to analyze the expression of all other Halloween genes, namely noppera-bo (nobo), spookier (spok), disembodied (dib), and shadow (sad), in the salm/salr RNAi PG. If it is confirmed that Spalt is indeed a transcription factor acting only on phtm, it will be recognized as a new transcription factor involved in the expression of the very specific subset of Halloween genes, such as ouija board, seance, and malting defective. This will be an important finding for the field of ecdysone biology.

-The regulation of Halloween genes is indeed a key aspect of Salm/Salr function. We have followed the reviewer advice and have now monitor the expression of Dib, Spok, Phtm, Nvd and Sro, for which we could obtain antibodies from Dr Michael O’Connor and Dr Ryosuke Niwa. We have made all these experiments in larvae carefully staged to make all the observations comparable, and the results are shown in the revised Fig. 5. We confirmed the reduction in Phtm expression and the lack of effects on Sro levels of expression. We also found that dib, spo and nvd expression is reduced in *salr-RNAi* PG. In the case of Nvd there is a discrepancy between our previous experiments and the new ones, because previously we could not detect effects on Nvd expression. We think this discrepancy is due to the staging of the larvae, because before we included L3 larvae of different ages and now we only used 6 days old larvae in all experiments. This was our mistake and we apologize for any misunderstanding. Unfortunately, we could not obtain antibodies for Nobo and Sad. These new results now indicate that there is a general reduction in the expression of most Halloween genes in *sal* mutant PC. Importantly, we are still very reticent to consider these observations as a demonstration of a function of Sal proteins as direct activators of Halloween gene expression, because Sal proteins are transcriptional repressors. As we now have more solid data concerning the relationship between Sal function and ERK activation, we interpret the effects of *salri* and *salmi/salri* knockdowns on Halloween expression as a consequence of reduced ERK activation.

-The changes we have made to correct previous results and to accommodate the new results are:

Page 6, corrected to: “*and lower levels of Phantom (Phtm), Spookier (Spok), Disembodied (Dib) and Neverland (Nvd) protein expression.*”

Page 14, modified heading to: “*The expression of Halloween genes is reduced in salr knockdown prothoracic glands*”

Page 14, modified to:
a) “We studied in control and in salr knockdowns PGs the expression of disembodied (dib), spook (spo), neverland (nvd), shroud (sro) and phantom (phtm) (Fig. 5). The expression of Dib (Fig. 5A-B, O), Spo (Fig. 5C-D, O), Nvd (Fig. 5G-H, O) and Phtm (Fig. 5I-J, O) is reduced in 7 days old larvae of phtm-Gal4 UAS-GFP/UAS-salr-RNAi PG compared to their controls phtm-Gal4 UAS-GFP/+, and only the expression of Sro is not modified in salr knockdown PG compared to their controls (Fig. 5E-F, O).”

b) “We tried rescue experiments by expressing nvd (UAS-nvd-HA/tub-Gal80ts; phtm-Gal4/UAS-salr-i) or phtm (UAS-phtm/tub-Gal80ts; phtm-Gal4/UAS-salr-i) in salr-i knockdown larvae. In the case of nvd we did not find a rescue of pupariation in salr-i larvae (32% of pupae compare to 26% in tub-Gal80ts; phtm- Gal4/UAS-salr-i). In contrast,….”

(3) The mitotic phenotype of the imaginal disc described in Figure 3 is also interesting. The must investigate whether this mitotic phenotype can be reversed by feeding with 20E.

© 2024. Published by The Company of Biologists under the terms of the Creative Commons Attribution License (https://creativecommons.org/licenses/by/4.0/).

The mitotic phenotype of imaginal discs deficient for ecdysone production of *phtm-Gal4/UAS-Smt3-RNAi* genotype is rescued by feeding the larvae with ecdysone (Herboso et al., 2015; Supple. Fig. 7; doi: 10.1038/srep12383). We have confirmed that the mitotic phenotypes caused by loss of *salm/salr* in the PG are also rescued by ecdysone in the food (Supple. Fig. 1).

We have made the following change to the manuscript:

-Page 13, modified to: "*We found that 20HE supplemented in the fly food (0.5-1 mg/ml) rescues cell proliferation in the wing disc and promotes the pupariation of UAS-salm-i/tub-Gal80ts; phtm-Gal4/UAS-salr- i (90%; Supple. Fig. 1 and Fig. 4M-N) and also promotes pupariation of phtm-Gal4 UAS-GFP/UAS-salr-i larvae (100%). salm/salr knockdown larvae forced to enter into pupariation by exogenous ecdysone die soon after they form the pupal case, as they are no longer supplemented with ecdysone (Fig. 4N).*

(4) The reviewer simply cannot understand the necessity of the data described in P15 and Fig.7. What is said in these data is not related in any way to the functionality of Spalt.

We believe these data are related to the functionality of Spalt because they show that reducing the expression of several components of the nuclear envelope results in cellular consequences and larval phenotypes similar to those caused by reductions in *Spalt* function. We have inserted a new paragraph that should help clarify the connection between Lamin/emb/Fs(2)Ket and Spalt:

Added, page 16: "*To search for similarities between the effects of salm/salr loss of function conditions in the PG on nucleolar size and chromosomal organization, we characterized the consequences of loss of function conditions in the PG of genes directly related with the organization of the nuclear envelope.*"

[Minor concerns]
(i) These days, the reviewer believes that the "data not shown" statement is basically inappropriate. In this manuscript, P9-L23, P10-L19, P12-L24, and P13-L12 have "data not shown". The reviewer believes that the manuscript should be revised to properly present these data as Supplementary Information.

-We have removed the "data not show" to either show a reference of state the result of the experiment:

P9-L23: Reference
P10-L19: Consistently, ectopic expression of Nvn (UAS-Nvn/tub-Gal80ts; phtm-Gal4/UAS-salr-i) fails to rescue pupariation in salr knockdown larvae (32% of pupae compare to 26% in tub-Gal80ts; phtm-Gal4/UAS- salr-i).
P12-L24: Deleted, as we stated that 54% formed smaller and 46% form larger than wild type larvae.
P13-L12: and also promotes pupariation of phtm-Gal4 UAS-GFP/UAS-salr-i larvae (100%).
(ii) P3, L17-18: "These proteins belong to the cytochrome P450 family of enzymes with monooxygenase activity": This sentence does not accurately describe the Halloween genes. Some Halloween genes, such as Nobo, Nvd, and Sro, do not encode P450 but other types of enzymes (GST, Rieske, and SDR, respectively). The authors need to revise this explanation.

Changed to:

Page 3, modified: "*These proteins display monooxygenase activity (Phamton, Disembodied, Shadow (Feyereisen, 2012), and other enzymatic activities such as Glutation transferase (Noppera-bo; Enya et al., 2014), Rieske electron carrier (Neverland; (Yoshiyama et al., 2006) and short-chain dehydrogenase/reductase (Shroud; (Niwa et al., 2010), and are encoded by a group of genes named the Halloween genes (Rewitz et al., 2006)(Gilbert et al., 2009).*

(iii) P6: The authors must describe a source of UAS-nvd used in this study.

Page 7, added: *UAS-nvd-Dm-HA* (Yoshiyama-Yanagawa et al., 2011)

© 2024. Published by The Company of Biologists under the terms of the Creative Commons Attribution License (https://creativecommons.org/licenses/by/4.0/).

(iv) P10, L2-3: While the authors mention the knockdown efficiency for salm-RNAI, somehow they don't mention the efficiency for salr-RNAI. The authors need to clarify this point.

-We haven´t measure the efficiency of the Salr-RNAi strain, because the anti-Salr antibody is not available anymore.

(v) P10, L19-20 and Fig. 1D, G: Based on the description in the manuscript, it is unclear whether the authors performed a temperature shift experiment with tub-Gal80ts/UAS- salm-i; phtm-Gal4/UAS-salr-I to obtain the larval phenotype or not. Was the experimental condition the same as that described by the authors at P11, L18? Please clarify.

-We added the following paragraph to Material and Methods section.

Page 7, added: "*Knockdown of sal genes in combination with the phtm-Gal4 driver were generated in the following genotypes: UAS-salm-RNAi/+; phtm-Gal4 UAS-GFP/UAS-salr-RNAi (salm-i/salr-i), UAS-salm- RNAi/+; phtm-Gal4 UAS-GFP/+ (salm-i), phtm-Gal4 UAS-GFP/UAS-salr-RNAi (salr-i) and tubGal80ts/+; UAS-salm-RNAi/+; phtm-Gal4 UAS-GFP/UAS-salr-RNAi (Gal80ts salm-i/salr-i). This last genotype was grown 2 d at 25º C, 5 d at 17º and 5 days at 29º C before larval dissection.*"

(vi) P11, L4: "Fig. 2A-F" should read "Fig. 2A-D, F". Alternatively, authors should switch between the current Fig. 2E and Fig. 2F.

-Done

(vii) P12-L12-13: Why did the authors use salr RNAi here instead of salm and salr double RNAi? The reviewer does not see any particular reason.

-We tend to use the single salr-RNAi instead of the double salm/salr RNAI because the former allows for the development of L3 with morphologically normal PC.

(viii) P12, L22-P13, L3 and Figs. 4M, 4N: The reviewer would like to see a graph showing the results of an analysis using a larger number of larvae to confirm what percentage of larvae are rescued into pupae by feeding on 20E.

-We have inserted in Figure 4N the percentage of larvae rescue to enter pupariation in the two genotypes analyzed. These values are also described in the text:

Page 14, modified: "*promotes the pupariation of UAS-salm-i/tub-Gal80ts; phtm-Gal4/UAS-salr-i (90%; Fig. 4M-N) and also promotes pupariation of phtm-Gal4 UAS-GFP/UAS-salr-i larvae (100%).*"

(ix) P13, L12-16: It is better to include the immunostaining data of the anti-Phtm antibody along with Figures 5E-H. The rabbit anti-Phtm antibody has already been generated by Michael B. O'Connor, Minnesota, USA.

-We have followed the reviewer advice and have included the data of anti-Phtm staining together with the previous *in situ* and b-Gal expression analysis of Phtm. In the new Fig 5 we also included new expression analysis and quantifications of Dib, Spo, Sro and Nvd using the corresponding antibodies.

(x) P13, L11: "Nvn" should be corrected to "nvd" (italicized).

-Done

(xi) P14, second paragraph, and Fig. 6C-F: For the general reader, please indicate with arrows, arrowheads, or other markers which are DNA, nucleoli, and nuclear envelopes, and also clarify these points in the figure legend.

-Done

© 2024. Published by The Company of Biologists under the terms of the Creative Commons Attribution License (https://creativecommons.org/licenses/by/4.0/).

(xii) P17, L11: "Prothoracic gland" should be abbreviated to "PG".

-Done

(xiii) In almost all figures, the blue letters of "DAPI" are barely visible. The authors need to consider a better way to show them.

-Done, changed color of DAPI to white in all Figures.

(xiv) Fig. 1A, 1B" On the figure, it is better to describe the genotypes used by the authors.

-Done

(xv) Figure 1F, 1G: On the figure, it is better to describe "phtm-Gal4" as the driver used.

-Done

(xvi) Fig. 2D-F: Can the authors show any counterstaining data to make sure that the PGs are present in these photographs?

-We changed the D-F panels in Fig 2 to show the expression of GFP. We also changed the G-K panels of Fig. 3 to show better images.

(xvii) As listed below, authors should properly cite some references in the manuscript. P4, L3-5: The authors should also mention Pvf and Jelly Belly, and also cite Pan and O'Connor, Cell Reports 2021.

-We inserted this and other refence concerning the role of RAS/ERK signaling (Pan and O'Connor, 2021 and Cruz et al., 2020).

P4, L10, and P12-L21-22: Instead of Niwa & Niwa 2016, authors should cite Komura-Kawa et al., PLOS Genetics 2015.

-Done

P5, L17: In addition to Danielsen et al. 2014, the following article should also be cited as this paper also focuses on ventral vein absence/drifter. Yunpo Zhao, Bo Gustav Lindberg, Shiva Seyedoleslami Esfahani, Xiongzhuo Tang, Stefano Piazza, Ylva Engström (2021) Stop codon readthrough alters the activity of a POU/Oct transcription factor during Drosophila development. BMC Biol 19(1):185. doi: 10.1186/s12915-021-01106-0.

-Done

P12-L12, In addition to Shirai et al. 2012, the following article should also be cited. Christian F Christensen, Takashi Koyama, Stanislav Nagy, E Thomas Danielsen, Michael J Texada, Kenneth A Halberg, Kim Rewitz (2020) Ecdysone-dependent feedback regulation of prothoracicotropic hormone controls the timing of developmental maturation. Development 147(14): dev188110. doi: 10.1242/dev.188110.

-In this paragraph we refer to the induction of EcR expression in the PG in response to 20HE, whereas the manuscript indicated by the reviewer analyses the induction of PTTH by EcR in PTTH-expressing neurons. In this case we have decided not to follow the reviewer suggestion.

P14-L5: The reviewer believes that "including Kni and Vvl (Danielsen et al. 2014)" should be deleted. Instead, it is better to cite Kamiyama & Niwa Frontiers in Physiology 2022 right after "other transcription factors".

-Done

© 2024. Published by The Company of Biologists under the terms of the Creative Commons Attribution License (https://creativecommons.org/licenses/by/4.0/).

Second decision letter

MS ID#: DEVELOP/2024/202751

MS TITLE: Developmental analysis of Spalt function in the Drosophila ring gland

AUTHORS: Cristina M Ostale, Diego Pulido, Patricia Vega-Cuesta, Ana Lopez-Varea, and Jose F. de Celis

I have now received all the referees reports on the above manuscript, and have reached a decision. The referees' comments are appended below, or you can access them online: please go to BenchPress and click on the 'Manuscripts with Decisions' queue in the Author Area.

The overall evaluation is positive and we would like to publish a revised manuscript in Development, provided that the referees' comments can be satisfactorily addressed. Please attend to all of the reviewers' comments in your revised manuscript and detail them in your point-by-point response. If you do not agree with any of their criticisms or suggestions explain clearly why this is so. If it would be helpful, you are welcome to contact us to discuss your revision in greater detail. Please send us a point-by-point response indicating your plans for addressing the referees' comments, and we will look over this and provide further guidance.

Reviewer 1

Advance summary and potential significance to field

I appreciate the authors' efforts to rework this manuscript, and also appreciate the addition of new data and graphs to this work. This has clarified several points for me, and I agree with the authors that it has improved the work.

Comments for the author

I suggest that the authors invert the storyline. Currently, the introduction starts with the prothoracic gland and how it synthesises the steroid hormone ecdysone. Then, the authors introduce Spalt proteins and their function as transcriptional repressors via interactions with chromatin and make the argument that these proteins might be involved in steroid synthesis. I think I would find the narrative more convincing if it was pitched in the opposite manner:
1) Spalt proteins are important transcriptional repressors that interact with heterochromatin to perform their function
2) We have a poor understanding of why these interactions are important.
Maybe this is because these proteins are studied mostly in diploid cells where chromatin state is difficult to visualise (the authors would know this better than I do)?
3) The prothoracic gland cells are a great system to understand the relationship between Spalt proteins and chromatin because they are polyploid.
Further, this gland has a well-defined function in the synthesis of ecdysone.
This is convenient because the phenotypes associated with interfering ecdysone production has been well described. Further, the genes involved in ecdysone synthesis are known providing the opportunity to understand the link between chromatin stability and gene expression.
In this way, the PG and its function are more of a convenient tool to test hypotheses about genome architecture and gene expression. If the paper is pitched as a story about ecdysone synthesis, the reader is left with the impression at the end of the paper that the PG cannot produce ecdysone because the cells are dysfunctional / dying.

If the authors decide to alter the storyline as suggested above, I would also change the focus of the title.

Please provide the methodology for the mouth hook dissection.

© 2024. Published by The Company of Biologists under the terms of the Creative Commons Attribution License (https://creativecommons.org/licenses/by/4.0/).

This sentence on page 13 doesn't make sense to me: "We found that the imaginal discs of late L2 UAS-salm-i /+; phtm-Gal4 UAS-GFP/UAS-salr-i (25C) are much smaller than wildtype wing discs from larvae of the same age, and similar in size to early L3 wildtype discs (Fig. 3A)." How can the L2 wing discs of the transgenic lines be smaller than L2 controls, yet the same size as early L3 wing discs of the controls?

Does overexpression of phtm in the phtm Gal4>salr-i rescue the nuclear phenotypes? If it doesn't, it would suggest that the wild type nuclear morphology isn't necessary for ecdysone production.

Minor comments:
1) Many of the paragraphs are really long, making them hard to follow.
Breaking these up into smaller paragraphs, with a single idea per paragraph would make the text easier to read.
2) In the results text, explain why you switch to using the tub-Gal80ts/UAS-salm-i; phtm-Gal4/UAS-salr-i genotype when you first introduce it.
This might not be obvious to people who don't work on ecdysone signalling or in Drosophila.
3) Could you reference the figure that supports this claim: "We found that 20HE supplemented in the fly food rescues cell proliferation in the wing disc
(20HE of 0.05mg/ml)…" ?

I've added minor edits to the pdf as well.

Note from editorial staff: These can be found in the supplemental file area labelled 'Reviewer 1 - minor corrections'

<u>Reviewer 2</u>

Advance summary and potential significance to field

In the manuscript, Ostale et al. investigate the role of Spalt transcription factors in the development and function of the prothoracic gland (PG) in the fruit fly Drosophila melanogaster. The study shows that the Spalt proteins Salm and Salr are required for ecdysone synthesis in the PG and are important for regulating the expression of several Halloween genes including phantom (phtm). Furthermore, the manuscript shows that loss of spalt function in the PGs results in abnormalities in nuclear organization, including chromatin distribution, nucleolus size, and nuclear envelope morphology. These abnormalities are associated with impaired ERK signaling, likely due to perturbations in nuclear architecture that affect nuclear pore function.
Over the past decade, transcriptional regulatory networks controlled by multiple regulators of transcription, chromatin remodeling, and endoreplication have been shown to be essential for the spatiotemporal expression control of Halloween genes in Drosophila melanogaster. However, it is still unclear how each of the Halloween genes is transcriptionally regulated in a spatiotemporally specific manner. In this context, this study successfully adds the new regulator for the PG function. In addition, the study also elucidates the role of Spalt in coordinating nuclear organization within the PG. This finding potentially sheds light on the role of intracellular architecture in ecdysone biosynthesis which is something new in this field of research.

Comments for the author

The reviewer feels that the revised manuscript is much improved. However, to be honest, I am still conflicted about whether to accept this revised manuscript. On the one hand, the authors have responded very sincerely and carefully to the reviewer's criticisms, performed many additional experiments, and added many new data. In particular, the comprehensive analysis of the Halloween enzyme, the overexpression experiment of Spalt, and the new data on the relationship with Erk can be evaluated as making the claims of this paper more certain. On the other hand, if one asks whether the contents of this paper provide any significant novelty regarding the control of the prothoracic gland, the reviewer does not think there has been much progress in understanding the mechanism of ecdysone synthesis and the physiology and development of the prothoracic

© 2024. Published by The Company of Biologists under the terms of the Creative Commons Attribution License (https://creativecommons.org/licenses/by/4.0/).

glands. The reviewer would like to reiterate that the authors' attitude in this revision was extremely sincere. I would like to leave the final decision to the editor.

Second revision

Author response to reviewers' comments

Response to Reviewer 1

1) I suggest that the authors invert the storyline. Currently, the introduction starts with the prothoracic gland and how it synthesises the steroid hormone ecdysone. Then, the authors introduce Spalt proteins and their function as transcriptional repressors via interactions with chromatin and make the argument that these proteins might be involved in steroid synthesis. I think I would find the narrative more convincing if it was pitched in the opposite manner:
1) Spalt proteins are important transcriptional repressors that interact with heterochromatin to perform their function
2) We have a poor understanding of why these interactions are important. Maybe this is because these proteins are studied mostly in diploid cells where chromatin state is difficult to visualise (the authors would know this better than I do)?
3) The prothoracic gland cells are a great system to understand the relationship between Spalt proteins and chromatin because they are polyploid. Further, this gland has a well-defined function in the synthesis of ecdysone. This is convenient because the phenotypes associated with interfering ecdysone production has been well described. Further, the genes involved in ecdysone synthesis are known providing the opportunity to understand the link between chromatin stability and gene expression. In this way, the PG and its function are more of a convenient tool to test hypotheses about genome architecture and gene expression. If the paper is pitched as a story about ecdysone synthesis, the reader is left with the impression at the end of the paper that the PG cannot produce ecdysone because the cells are dysfunctional / dying.

We have changed the story line according to the reviewer preference. After making this change, we have to agree with the reviewer that it makes sense to start describing the Sal proteins and then the PG and ecdysone synthesis. This suggested storyline is also more aligned with the Abstract.

2) If the authors decide to alter the storyline as suggested above, I would also change the focus of the title.

We have decided to retain the original title, as we think it is a trustworthy reflection of the manuscript message.

3) Please provide the methodology for the mouth hook dissection.

We have inserted the following paragraph to the material and methods section:

Page 7, inserted:
Mouth hook dissection
The mouth hooks were dissected in water removing with tweezers the tissues attached to then. After dissection, the mouth hooks were mounted in 70 % glycerol in microscopic slices. The cover slides were sealed with nail polisher, and the preparations photographed in a Zeiss Axioplan microscope using a Spot 2 digital camera."

4) This sentence on page 13 doesn't make sense to me: "We found that the imaginal discs of late L2 UAS-salm-i /+; phtm-Gal4 UAS-GFP/UAS-salr-i (25C) are much smaller than wildtype wing discs from larvae of the same age, and similar in size to early L3 wildtype discs (Fig. 3A)." How can the L2 wing discs of the transgenic lines be smaller than L2 controls, yet the same size as early L3 wing discs of the controls?

© 2024. Published by The Company of Biologists under the terms of the Creative Commons Attribution License (https://creativecommons.org/licenses/by/4.0/).

The reviewer is right, as we are comparing L2 discs of arrested salmi/salri larvae with normal early L3 discs. We have changed the sentence to:

Page 12: "We found that the imaginal discs of arrested late L2 UAS-salm-i /+; phtm-Gal4 UAS-GFP/UAS-salr-i larvae (25 °C) reach a maximal size similar to that of early L3 wildtype discs (Fig. 3A, C-D)."

5) Does overexpression of phtm in the phtm Gal4>salr-i rescue the nuclear phenotypes? If it doesn't, it would suggest that the wild type nuclear morphology isn't necessary for ecdysone production.

This is a very interesting point, because we are assuming that nuclear morphology is a direct result of sal loss that leads to reduced ERK signaling and consequently reduced transcription of Hallowing genes. In this scenario, we expect that the rescue by UAS-phtm of the phtm Gal4>salr-i phenotype occurs without affecting the nuclear envelope.

We don't agree that such a result implies that "nuclear morphology isn't necessary for ecdysone production", as this is only correct in a situation where we are providing phtm expression independently its normal regulatory mechanisms, and therefore independent of the sal mutant effects on nuclear morphology.

Coming back to the reviewer question, we did not look at nuclear morphology in the UAS-phtm rescue of the phtm Gal4>salr-i phenotype. However, we have looked at nuclear morphology in the UAS-Ras1V12 rescue experiments, and the result is that in this context, which increases the efficiency of ERK activation independently of sal, the nuclear morphology is the characteristic of loss of sal function, as we expected.

We added the following sentence:

Page 18: "without affecting the increase in nucleolar size characteristic of salr mutant PG (Fig. 8M, O)."

6) Minor comments:
1) Many of the paragraphs are really long, making them hard to follow. Breaking these up into smaller paragraphs, with a single idea per paragraph, would make the text easier to read.

We have split the following paragraphs in the result and discussion sections:

Page 11
Page 13
Page 17
Page 18
Page 19
Page 21

2) In the results text, explain why you switch to using the tub-Gal80ts/UAS-salm-i; phtm-Gal4/UAS-salr-i genotype when you first introduce it. This might not be obvious to people who don't work on ecdysone signaling or in Drosophila.

We have modified a sentence that should explain this:

Page 11: "When we reduced the efficiency of the double salm/salr knockdown, by using the P0206-Gal4 and amnC651-Gal4 drivers or the tub-Gal80ts construct,…."

3) Could you reference the figure that supports this claim: "We found that 20HE supplemented in the fly food rescues cell proliferation in the wing disc (20HE of 0.05mg/ml)…" ?

We inserted the reference to Supple. Fig. 1 in page 14:

© 2024. Published by The Company of Biologists under the terms of the Creative Commons Attribution License (https://creativecommons.org/licenses/by/4.0/).

“We found that 20HE supplemented in the fly food rescues cell proliferation in the wing disc (0.05mg/ml 20HE; Supple. Fig. 1),…”
4) I’ve added minor edits to the pdf as well.
Done

Third decision letter

MS ID#: DEVELOP/2024/202751

MS TITLE: Developmental analysis of Spalt function in the Drosophila ring gland

AUTHORS: Cristina M Ostale, Diego Pulido, Patricia Vega-Cuesta, Ana Lopez-Varea, and Jose F. de Celis

I am happy to tell you that your manuscript has been accepted for publication in Development, pending our standard publication integrity checks.

© 2024. Published by The Company of Biologists under the terms of the Creative Commons Attribution License (https://creativecommons.org/licenses/by/4.0/).
